# Singing Together, Yet Apart: The Experience of UK Choir Members and Facilitators During the Covid-19 Pandemic

**DOI:** 10.3389/fpsyg.2021.624474

**Published:** 2021-02-18

**Authors:** Helena Daffern, Kelly Balmer, Jude Brereton

**Affiliations:** ^1^AudioLab, Department of Electronic Engineering, York Centre for Singing Science, University of York, York, United Kingdom; ^2^In2Voice, York, United Kingdom

**Keywords:** singing, choirs, virtual choirs, wellbeing, multi-track

## Abstract

The Covid-19 induced United Kingdom-wide lockdown in 2020 saw choirs face a unique situation of trying to continue without being able to meet in-person. Live networked simultaneous music-making for large groups of singers is not possible, so other “virtual choir” activities were explored. A cross sectional online survey of 3948 choir members and facilitators from across the United Kingdom was conducted, with qualitative analysis of open text questions, to investigate which virtual choir solutions have been employed, how choir members and facilitators experience these in comparison to an “in-person” choir, and whether the limitations and opportunities of virtual choir solutions shed light on the value of the experience of group singing as a whole. Three virtual choir models were employed: Multi-track, whereby individuals record a solo which is mixed into a choral soundtrack; Live streamed, where individuals take part in sessions streamed live over social media; Live tele-conferencing, for spoken interaction and/or singing using tele-conferencing software. Six themes were identified in the open text responses: Participation Practicalities, encompassing reactions to logistics of virtual models; Choir Continuity, reflecting the responsibility felt to maintain choir activities somehow; Wellbeing, with lockdown highlighting to many the importance of in-person choirs to their sense of wellbeing; Social Aspects, reflecting a sense of community and social identity; Musical Elements, whereby the value of musical experience shifted with the virtual models; Co-creation through Singing, with an overwhelming sense of loss of the embodied experience of singing together in real-time, which is unattainable from existing virtual choir models. The experiences, activities and reflections of choir singers during lockdown present a unique perspective to understand what makes group singing a meaningful experience for many. Co-creation through Singing needs further investigation to understand the impact of its absence on virtual choirs being able re-create the benefits of in-person choirs.

## Introduction

There is now a firmly established and rapidly growing body of research which considers the value of group singing, especially in terms of potential health and wellbeing benefits. Singing together seems to be a highly valuable experience for many individuals, with much of the work to date contributing to a scoping review of the evidence on the role of the arts in improving health and wellbeing (Fancourt and Finn, 2019). Research reviewed by Clift et al. (2008) indicates that group singing is a valuable activity, but revealed the need for a better understanding of the connections between the physical, psychological, and social processes. Later qualitative studies observed social and mental wellbeing benefits of choir singing (e.g., Clift et al., 2010; Skingley and Bungay, 2010; Livesey et al., 2012; Judd and Pooley, 2014) and a randomized control trial found group singing to have a positive impact on affect and anxiety, but not on physiological indicators (Sanal and Gorsev, 2014). The United Kingdom-wide lockdown in 2020 in response to the Covid-19 pandemic saw in-person choir activity halted and choirs looking to virtual alternatives to try to maintain their activities. This provides a unique perspective from which to consider how different elements of choir singing contribute to the perceived benefits once face-to-face contact is lost.

In considering why group singing is found to be beneficial, across all populations and study types, the social elements of the activity contribute substantially to the reported benefits. Gick (2011), highlighting the need for more research, reviewed studies on singing as it relates to health and wellbeing, finding that positive outcomes for social factors were only associated with group singing. Comparing choir singing with solo singing and team sport, questionnaire responses indicated that solo singing resulted in significantly lower reported wellbeing, but with choir singing creating a more coherent “meaningful” social group than the sport activity (Stewart and Lonsdale, 2016). A recent study of Scandinavian choirs forced to stop activities during a societal partial lockdown in the spring of 2020 highlights further the importance of the social elements of the activity. Participants reported missing the social aspect more than the other six components that were assessed, which were related to aesthetic experience, flow, and physical aspects of singing (Theorell et al., 2020).

The reasons that strong social bonds are formed through interactions of musicians are complex and difficult to research, but it is likely that a number of factors interact, with group singing providing something “special” beyond mere social engagement such as conversations between members when they aren’t singing.

The importance of social context and resulting benefits of group singing have been considered within frameworks of social capital (Louhivuori et al., 2005), including the key indicator of fellowship within community choirs (Langston and Barrett, 2008). Tarr et al. (2014) suggest the association of “self-other merging” and neurohormonal mechanisms contribute to the strengthening of social bonds. Specker (2017) focuses on the role of the musical elements of the activity with sound creation and the reciprocal interaction and coordination of singing as a physical process as key to the social cohesion experienced. This links to her earlier work exploring the process of group singing as a form of communitas (Specker, 2014) and Turino’s definition of “sonic bonding” (Turino, 2008) whereby the physical coordination of music-making creates this social cohesion. Considering group singing as a complex adaptive system and the social bonding which that affords, Camlin et al. (2020) discuss group singing as a form of “healthy public,” drawing on participant stories of a community singing project to try to capture the multidimensionality of participants’ experiences.

If group singing is indeed a beneficial activity for participants, and more so than other “social” activities, the importance of the shared creation of music through the coordinated physical action of singing together within a shared physical space would indicate that current virtual choir solutions would not, and could not, replicate the benefits observed in “in-person” choir activities.

Virtual Choir is a term that has come to encompass any activity or output that is associated with choirs in which the members do not share a physical space during rehearsal or performance. Before the Covid-19 outbreak in 2020, virtual choirs were a novelty, perhaps the most famous being the Eric Whitacre’s virtual choir, which initially involved multi-tracking solo recordings and was later developed to include a real-time element, connecting performers across the globe (Whitacre, 2018). The aim was to bring singers and their love of music together utilizing emerging technologies in novel ways. Addressing the potential wellbeing benefits of Whitacre’s virtual choir, Fancourt and Steptoe (2019) conducted a study which assessed the perceptions of participants from Whitacre’s virtual choir project compared to participants of live choir rehearsals, focusing on questions centered around social presence, and emotion regulation. Those engaged in the Whitacre project self-rated social presence higher than matched singers in live choirs, the authors concluding that virtual experiences may be valuable for those who cannot engage in live choir experience.

In response to the United Kingdom-wide lockdown during the Covid-19 pandemic in 2020, Whitacre launched Virtual Choir 6: Sing Gently, which includes 40000 singers from 145 countries, and many other virtual choir initiatives were born, intended for both local, national, and international engagement. As “real life” choirs sought to find alternatives to no longer being able to meet, rehearse and perform in-person, available technology was used to try to satisfy the priorities of choirs across the globe. Not only did the lockdown situation firmly establish “virtual choir” within common terminology, its meaning came to encompass an increasingly diverse range of situations and outputs.

Within the recent plethora of “virtual choirs” is the uptake of the multi-track model used by Whitacre, whereby individuals record their own individual part and the sound files are edited together to create a choral recording. Two further models of live virtual choirs have since emerged that allow for some form of real-time interaction with other singers. The first is live-streamed sessions over social media such as Facebook, which encourage singers to sing along, karaoke-style, with a live stream and post live messages in the chat boxes. This format has especially been used as a means of bringing together new groups of singers without geographical boundaries.

The second employs existing video conferencing software, such as Zoom, to run choir meetings online, aiming for maintenance of choir routines such as regular rehearsals. In this setup, choir members are able to engage in spoken conversation, and/or chat via the chat boxes. Whilst all choir members are able to sing at roughly the same time, due to issues of latency it is necessary for everyone to have their microphones muted, except for one member (usually the facilitator) which means that the singers in the choir cannot hear each other whilst they are singing.

The popularity of virtual choirs in lockdown demonstrates a desire to regain, somehow, and to some extent, the in-person experience of group singing and its many extolled associated benefits (Burkeman, 2015). However, none of these models provide an experience of singing together in real-time whereby singers can hear and therefore react to, and interact with, each other. In terms of re-creating an in-person choir experience all current solutions “fail to replicate or simulate any aspects of live performance as a spatially, temporally situated act undertaken by embodied beings engaging in an immediate and intimate mode of co-creation” (Datta, 2020). The experiences offered by the various virtual choir models are very different from one another, and yet all virtual choirs offer *something* of the “choir” experience which is deemed valuable by those participating.

## Aims and Research Questions

The Covid-19 pandemic forced choirs to cease meeting in-person, and presents a unique opportunity to not only explore the social value of virtual choir experiences, but to also consider the wider impact on participants of their choir experience and their perceptions of their “choir” once defining elements of the activity are unattainable. This paper therefore aims to understand how individuals who usually engage in group singing activities have responded to the restrictions enforced by Covid-19, both in terms of practical engagement with virtual choir initiatives and self-reported emotional reactions to those initiatives.

The following research questions are addressed:

• What virtual choir solutions have been employed by United Kingdom choirs during the Covid-19 lockdown?
• How do members and facilitators feel the experience of the virtual choir solutions differs from their “in-person” choir?
• Do the limitations and opportunities of the virtual choir solutions shed light on the value of the experience of group singing as a whole?

## Method

This study was approved by the University of York’s Physical Sciences Ethics Committee (ref: Daffern030720). A cross-sectional online survey with multiple choice and open text questions was created and distributed within the United Kingdom using Qualtrics software (Qualtrics, Provo, UT, United States). The survey link was distributed to publicly available email addresses of choral associations and networks as well as individual choirs/vocal groups, which were collected in a separate internet audit of United Kingdom choirs to be reported elsewhere, and via social media. The questionnaire inventory and flowchart are provided in Supplementary Appendices 1 and 2, respectively. Lockdown in the United Kingdom began on 23rd March 2020 and the survey was active from 20th July 2020 to 4th August 2020.

An inductive approach was utilized for thematic analysis of open text responses whereby themes were not determined prior to analysis of the data (Thomas, 2006). After familiarizing themselves with the material, Daffern and Balmer created an initial codebook through joint analysis of a selection of responses. All responses were then coded independently by both authors in Qualtrics by manually assigning topics to responses. A “live” codebook table was kept in a Google Doc to which any additional codes were added and discussed throughout the process. Co-author Brereton then performed independent parallel coding on a 10% random sample of responses with survey questions removed, which were cross checked and assimilated with the coding of the other authors. Two theming workshops were then conducted with all three authors, during which the codes were categorized into subthemes and main themes generated from the coded data. The coding tree can be found in Supplementary Appendix 3. The main themes were assigned to the codes as Parent Topics in Qualtrics and reports generated to compare facilitator and member responses as well as multi-track vs. live choir models.

## Survey Responses

**FIGURE 1:**
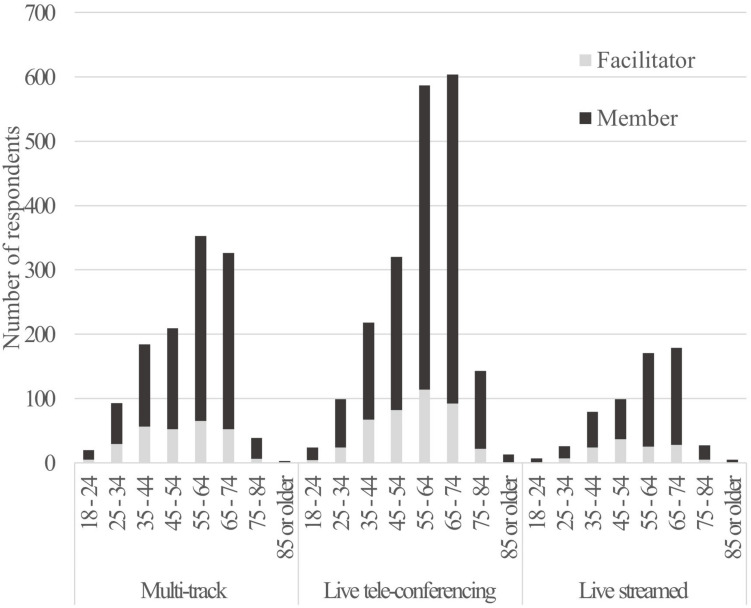
Showing the distribution of respondents by age, role (facilitator or member), and type of virtual models engaged with.

There were 3948 responses to the survey, with 819 respondents identifying as facilitators/conductors (male: 293; female:518; other: 7) and 2753 as choir members (male: 592; female:2144; other: 17). Out of the 2753 choir members, 823 (30%) reported that none of the choirs they normally sang with continued during the Covid-19 lockdown, of these 128 (5%) joined a new virtual choir opportunity that was not associated with their usual in-person choir. Those who did not engage in any virtual choir (17%) reported either having no virtual choir opportunities or citing reasons that align with the negative themes highlighted across all respondents. 296 (38%) of facilitators did not continue their choir online. Figure 1 shows the age distribution of all respondents by member and facilitator and type of virtual choir, with most responses within the 55–74 age bracket.

Table 1 shows the types of choirs respondents were part of before the Covid-19 pandemic.

**TABLE 1:** Types of choir engaged with by respondents before Covid-19.

Choir Type	% responses
Community Choir	36.1
Large Chorus Choir	20.6
Church Choir	13.5
Chamber Choir	11.4
Mixed	8.8
Pop Choir	5.1
Rock Choir	5.1
Male voice	4.5
Show Choir/Musical theater/G&S	2.8
Barbershop Choir	2.5
Workplace Choir	2.1
Solo Voice Ensemble	1.7
Folk Choir	1.4
Female voice	1.3
Therapeutic Choir	1.2
Gospel Choir	1.2
Bach Choir	1.2
Other (x32)	4.6

### Engagement With Virtual Choirs

The types of virtual choir that respondents engaged with during lockdown are summarized in terms of their practical implementation as three virtual models in Table 2. The engagement of respondents with the three virtual models is shown in Figure 1, with the Live tele-conferencing model most commonly used.

**TABLE 2:** A summary of the virtual choir models used during the lockdown of 2020.

	**Virtual choir models**		
	Multi track	Live streamed	Live tele-conferencing
**Output**	A “choral” recording (with or without video) created from mixing the solo tracks together.	• No recorded/choral output unless combined with the multi-track model	• No recorded/choral output unless combined with the multi-track model
**Facilitator**	**Pre-session** • Creating backing/template tracks • Make music copies available • Provide instructions for choir members • Organize uploading of recordings **During session** • *Ad hoc* troubleshooting for members making recordings **Post-session**: • Edit the video/audio tracks • Disseminate the video/audio tracks • Optional feedback coordination and reflection **Requirements** • Good technical skill needed to edit audio and video tracks and together • Video/Audio editing software • Good internet upload/download speeds to process large audio/video files • High quality audio interface and microphone for backing tracks **Scheduling** • Flexible, no requirement to create scheduled events. • Deadlines are needed for members to submit their individual contributions	**Pre-session** • Planning sessions • Checking technology works **During session** • Delivering the sessions • Conducting and/or playing accompaniment • Checking technology working appropriately **Post-session** • Optional feedback coordination and reflection **Requirements** • Minimal technical skill • Stable high-speed internet connection • High quality audio interface and microphone **Scheduling** • Needs to take place when advertised	**Pre-session** • Planning sessions (these often include non-musical social activities such as quizzes) • Checking technology **During session** • Running/hosting the sessions • Checking technology working appropriately • Potential for conducting and/or playing accompaniment **Post-session** • Optional feedback coordination and reflection **Requirements** • Minimal technical skill needed • Technology required • Stable internet connection • High quality audio interface and microphone **Scheduling** • Needs to take place when advertised
**Member**	**Pre-session** • Song learning • Technology set-up (following guidelines of the facilitator) • Checking technology works **During session** • Record a solo track to a pre-recorded backing track **Post-session** • Wait to hear results of final edit (sometimes weeks/months later) • Help to disseminate the result **Requirements** • Minimal technical skill • Headphones • Recording device • Hardware for playback of the backing track • Quiet space for recording **Scheduling** • No scheduled time for participation, just within the deadline for recording submission (unless combined with a live model for rehearsals) **Choir size** • Theoretically large numbers of individuals can take part • Practical limit on time available for audio editing • Practical limit of processing power of computer used for video editing	**Pre-session** • Sometimes song learning • Technology set-up • Checking technology works **During session** • Sing along to the live stream • Live interaction through chat messages **Post-session** • Potential to provide feedback **Requirements** • Only playback technology needed • Ideally a space at home to sing without disturbing others **Scheduling** • Scheduled time to join the live stream, but can play the recording at a later date **Choir size** • No restrictions on numbers taking part as one-way streaming only	**Pre-session** • Sometimes song learning • Technology set-up • Checking technology works **During session** • Sing along with others with your audio muted to a single channel of reference audio (e.g., the conductor) • Live interaction through real time conversation with two-way video and audio **Post-session** • Potential to provide feedback **Requirements** • 2-way technology needed (e.g., integral microphone for two way communication) • Good upload as well as download speeds • Ideally a space at home to sing without disturbing others **Scheduling** • Need to take part at the pre-scheduled time **Choir size** • Theoretically large numbers of individuals can take part • Practical limit to taking part in group chat activities, difficult with larger groups • Practically only small numbers of singers can sing with microphones unmuted*

*If using specialist software designed to enable low-latency audio over home internet connections (such as JackTrip), small groups should be able to perform together without the need for each participant to keep their microphone muted. Such technology depends on high speed internet connections (usually wired) and excellent computer skills for the initial setup for all users.

From here on in live virtual choir refers to both Live models (streamed and tele-conferencing) unless specified. Around half (187 = 46%) of the facilitators of a Live virtual choirs found that engagement waned over time whilst 186 = 47% reported steady engagement and 7% (29) an increase since starting the virtual choir. This is reflected in the responses of members, with 52% of Live virtual choir members taking part in all online sessions that were offered, whilst 33% only attended some sessions and the remaining members rarely or never attended online sessions.

For multi-track members, 51% took part in all the multi-track opportunities offered by their choir and 76% enjoyed watching the final version once it had been produced. Facilitators reported general feedback to the multi-track choir experience to be “mainly positive” (117 = 54%) “only positive” (62 = 28%) and “mixed” (34 = 16%).

## Thematic Analysis

Six main themes with subthemes were generated through the reflexive thematic analysis of the coded open text questions: Participation Practicalities, Choir Continuity, Wellbeing, Social aspects, Musical Elements, and Co-creation through Singing. Supplementary Appendix 4 presents the themes and subthemes with illustrative responses provided.

### Participation Practicalities

The overriding frustration across survey responses came from the inability of any virtual model to allow singers to sing together and hear each other in real-time. Although some facilitators became fond of the mute button on zoom (due to the cacophony created by the latency when attempting to sing together), there was a unanimous dislike of the inability to hear each other when singing.

Within the understood limits of the models being used, there were also many barriers to online participation stemming from lack of facility with technology, lack of suitable hardware, and the potential cost of purchasing suitable equipment.

For some, a personal lack of skill using the required technology, or a perception that the technology was unreliable limited their capacity to be involved and ultimately reduced their enjoyment of participating in a virtual choir.

…the additional fight with unreliable technology just makes an unsatisfactory experience a really stressful one [Female; 65–74; Member; All; Qu 24].

Some choir members did not own suitable computer hardware equipment or could not access the software required, and were not able to support the cost of purchasing equipment, countering perceived improvements to accessibility for those with the technology and skill.

A few singers don’t have access to IT so we’ve had to rely on dial-in connections, which restricts what I can do in rehearsals without excluding those members [Male; 45–54; Facilitator; All; Qu 46].

....getting online is not accessible to everyone, and can be very confronting to those who are older/technophobic [Female; 35–44; Facilitator; Live T; Qu 46].

Internet connections vary greatly across households and throughout the Country, and are slower and/or less reliable in certain areas.

Internet connection in this rural part of Suffolk/Essex has prevented us from doing choirs online – due to the varied delays [Female; 55–64; Facilitator; None; Qu 53].

Some choirs attempted to sing over conferencing software in real-time as a choir (without muting themselves) and resorted to only social gatherings.

We tried sin[g]ing only once, and it was a disaster because of everyone’s different bandwidths, so we now just use it as a social occasion [Female; 55–64; Facilitator; MT and Live T; Qu 39].

Those who took part in multi-track projects had mixed reactions to the process of recording the solo tracks and then putting together the multi-track recording from individual choir members’ contributions.

I really miss singing with others, how our voices blend in person, etc. But I have to say that the final products have all been really amazing! [Female; 45–54; Member; Both, Qu 19].

There was much additional effort required for both live online choirs, and multi-track recording experiences which for many dampened the otherwise positive experience of singing in a choir and performing “together.” For the facilitators this involved much more preparation time (and in the case of the multi-track model, often hours of post-production editing).

Although initially participating in a multi-track choir was often difficult at first, especially in terms of learning new technology, the task of joining online became easier with practice.

Although recording your part alone at home feels odd at first, the more you participate in, the easier it becomes [Female; 65–74; Member; Multi-track; Qu 19].

For some the virtual choir actually made participation easier than the in-person choir rehearsals; in comparison they found the virtual choir to be more convenient, more flexible, less time consuming, with less travel, and no weather considerations as well as often being less expensive.

Time and financial advantages. No travel time or expenses. No hire of hall fees. More flexible- can sit with a cup of tea, etc. Can join in to suit – not committed to the whole session if something else impending but able to take part in some of it rather than having to miss session (sic) [Female; 65–74; Facilitator; Live S and Live T; Qu 52].

Virtual rehearsals accessed from the choir member’s own home were also seen as more convenient in terms of time, flexibility around other commitments, the reduced need for childcare to allow the choir member to participate, avoiding travel during inclement weather and the possibility of arriving late or leaving early if needed to fit around work and family commitments.

Easier for many, as our Singers are Seniors and prefer not to travel, so attendance has been more consistent than meeting face to face, even before the pandemic [Female; 65-64; Facilitator; All; Qu 39].

Conversely, others had the difficulty of literally finding “space” at home from which to participate in an online choir without too many distractions.

Not easy to sing fully in a constrained home environment – disturbance to others, noises off and disruption [Female; 45–54; Member; MT; Qu 24].

There was also a strong sense of “zoom fatigue” from members as well as a reluctance to engage with yet more screen-based activities.

Virtual choir rehearsals and performances can be more accessible than their in-person counterparts, allowing those with access to the required technology the possibility of singing with others outside of their local community, facilitating a wider, even global perspective, to choral participation. Many participants noted that the virtual models opened up new possibilities to make connections with choirs and singers across the country, or even world-wide.

Can virtually meet new people from anywhere in the world [Female; 65–74; Facilitator; Live S and Live T; Qu 52].

It also allowed those who were “shielding” due to health concerns to continue to participate in their usual choir, or to join some of the virtual choir projects organized on a larger scale.

There is one experience which if it wasn’t for coved (sic) 19 I would never have had the opportunity to do, and that is to sing with Opera North Choral. Amazing [Female; 65–74; Member; Live S and Live T; Qu 21].

For one particular friend it provided a lifeline when she was shielding and all her other forms of social contact had been taken away [Female; 55–64; Member; Live T; Qu 53].

Whilst overwhelmingly, reference to economic associations of virtual choirs were negative related to the overall impact of Covid-19 restrictions and loss of funding and personal income, there were also economic advantages to the virtual choir experience, since an online choir can be accessible at lower cost than the usual face-to-face meeting, with reductions in travel costs, and little, or no, rehearsal venue hire costs. 285 members taking part in live virtual choirs and 170 involved in multi-track virtual choirs reported being asked for additional donations to maintain their choir during lockdown.

Although many members were saddened that fundraising initiatives and community performances were halted for their choirs, others continued and found these to be other positive rewards, often in the form of being able to contribute to the community by providing freely available recordings, or raising funds for charity.

We are about to release our second recording in support of a local charity and the confidence of members has improved enormously around doing this sort of thing. It was hard to convince them to do it on board but the results were worth it. It has been extremely time consuming but it has been a great exercise in learning about individual members and their voices. It has also been an extremely positive force for the choir, with our first attempt raising over €3000 for charity [Male; 25–34; Facilitator; Live S and Live T; Qu 31].

### Choir Continuity

There was a very strong sense amongst choir members that they wanted to try to maintain the choir during lockdown. The reasons were multiple, but often centered around wanting to retain some “normality” and wanting to have something to return to: many respondents referred to their virtual choir as useful as “*a stop gap*” but “*not a long term solution*.”

I am pleased to have taken part in my choir leader’s virtual choir so that I can keep in touch with her and other choir members. However, I am so looking forward to being able to meet normally [Female; 65–74; Member; Live; Qu 53].

The inherent limitations of existing video conferencing technology were well understood, and grudgingly accepted, by choir members and facilitators alike as the only means available with which to attempt to maintain some continuity.

Would love to be able to sing together and hear everyone. I understand that there is a slight delay and so if we’re not all muted on Zoom it would sound cacophonous [Female; 55–64; Member; Live T; Qu 22].

Although members saw several drawbacks to the virtual choir experience, there was a strong sense of willingness to put up with these difficulties and inconveniences by placing the virtual choir in the context of “doing something,” no matter how difficult or unsatisfactory, was better overall than “doing nothing.”

It is a pale substitute for the real thing but nonetheless important for morale, continuity and wellbeing [Female; 65–74; Member; All; Qu 21].

Sometimes this desire for continuity is underpinned by financial motivations. Such financial concerns were manifold.

The general or longer term financial viability of the choir was often a consideration, often looking forward to making sure that a return to “normal service” would be economically feasible for the choir once the global pandemic and lockdown restrictions had abated.

Long term, our financial viability is a concern [Female; 55–64; Mt and Live T; Qu 53].

I am concerned for the numerous smaller community choirs around the country who may now be in dire financial straits and will not still be there [Female; 25–34; Member; All; Qu 53].

For some choirs, the choir facilitator had continued in order to maintain a personal income stream, and similarly the participants had continued in support of their conductors income. In addition for choirs where members pay fees to participate, facilitators felt a sense of responsibility to provide what had been paid for.

I also “owed” some sessions to members who had Pre paid for the term, and didn’t want to refund them so had to keep something going. Also. I was stubborn and didn’t want it to get the better of everything I had built up over the past 5 years! [Female; 35–44; facilitator; All; Qu 53].

Similarly choirs also have a sense of responsibility toward the charities that they are used to supporting through fundraising concerts and other activities, and a frustration that these normal activities have been curtailed, potentially impacting the wider community, outside of just choir members and their families.

Frustration that we cannot raise money for charities from our concerts [Female; 65–74; Facilitator; Live T; Qu 53].

Quite striking was the very strong sense of responsibility felt by many individual participants and facilitators toward each other, together with a sense of loyalty toward each other and the choir as an organization.

“I felt a sense if (sic) responsibility to each choir to continue to provide content and create social get togethers remotely” [Female; 45–54; facilitator; Live T; Q 44].

“Sometimes don’t feel like ‘bothering’ to join the community choir zoom group – but usually do out of loyalty” [Female; 65–74; member; Live T; Q 53].

This sense of responsibility and loyalty toward their choir was often the driving force behind participation when motivation was lacking, especially maintaining motivation to engage with the virtual choirs as lockdown continued.

My members have found online helpful and it has given them motivation and something to look forward to, I have found it hard and frustrating, but have continued to do it because it has helped so many ladies in this difficult time. They haven’t even wanted to stop for the summer! [Female; 55.64; Facilitator; Live T; Qu 53].

For other choirs, it was imperative to try to sustain the current membership of the choir, and potentially to even grow the choir membership, by exploiting the benefit of technology that meant that some barriers to participation were ameliorated for example for those who previously were unable to attend in-person rehearsals.

### Wellbeing

Although within the context of lockdown virtual choirs were often seen as a “lifeline” toward maintaining wellbeing, respondents also reported that the lack of in-person choir meetings and rehearsals meant that the usual contribution toward choir members’ wellbeing was not provided; the lack of that regular time during the week, where singing together could act as a kind of group meditation, an immersive activity that many saw as helping to reduce day-to-day stress and improve their energy levels, was sorely felt. The virtual alternative was generally seen as a poor substitute for the psychologically uplifting experience of singing together in the same space which they had previously relied upon.

I didn’t realize how important my choir was to my wellbeing and sense of identity [Female; 35–44; Member; All; Qu 53].

Now its gone I’m realizing just how much choir affected my mental and spiritual well-being Quire singing is an important part of my life; it helps me feel alive, so not being able to practice together properly is quite destructive of my overall sense of wellbeing [Male; 53–64; Member; Live T; Qu 53].

Often linked to the maintenance of social connections between members, there is a strong sense of virtual choirs providing a “lifeline” during unique circumstances, providing a sense of purpose and connection for individuals.

Maintains social contact and wellbeing for some potentially isolated older members [Male; 55–64; Member; Live T; Qu 56].

I suppose you can feel part of something bigger, which contributes to a sense of well-being [Female; 55–64; member; Mt and Live T; Qu 23].

Although the social connections were often connected with wellbeing, choir members still felt uplifted from the activity of singing, even though it was aurally a solo activity in both virtual models.

I would rather have this opportunity than nothing. Singing is my therapy. It is something I could not imagine doing without [Female; 65–74; Member; MT and Live T; Qu 23].

Without the virtual choir, I would have found it much harder. The singing helped keep me on an even keel, I felt more relaxed after each session [Female; 55–64; MT and Live T; Qu 53].

For some, participating in a virtual choir in itself actually had a strong negative impact on wellbeing Rather than simply feeling that “something was missing” that prohibited their usual enhanced sense of wellbeing, participating virtually was actually detrimental compared to not participating at all, eliciting feelings of stress and sadness.

I really wanted to join in with online choirs but then the idea of singing alone to a screen really emphasized just how to much life had changed due to Covid. So it made me sad rather than happy [Female; 65–74; Member; None; Qu 26].

For some choir members the lack of in-person rehearsals meant that they had lost a key activity that they had participated in either wholly or partly to maintain good mental health, just at the very time that mental health had been very adversely affected due to the impact of a global pandemic and lockdown situation. The lack of choral activity as a key part of maintaining good mental health was very keenly felt, and often interwoven with despair and anxiety arising from the lockdown situation.

…basically my mental health has crashed and with no idea when we might be able to get together again I am losing hope [Female; 45–54; Facilitator; All; Qu 53].

Virtual choirs, whilst enabling some interaction actually increased a sense of loneliness for some participants, highlighting how much of the social aspect of being in a choir they were actually missing.

It can feel a bit lonely, especially when you say goodbye and click “leave” And I’m singing alto on my own, which is weird (and freaks the cats out!) [Female; 55–64; Member; Live T; Qu 24].

The Covid-19 lockdown situation seems to have highlighted, for members and facilitators alike, a heightened awareness of the specific importance of the social aspect of choir membership, which is often intertwined with the positive wellbeing benefits of choral singing.

### Social Aspects

When considering differences between virtual and in-person choir, *missing social aspects* and *social interaction* was highlighted particularly in the multi-track model, with Live virtual choir members commenting less on social aspects here. However, when asked specifically to identify any advantages or disadvantages to the virtual choirs, the members in both choir types reported both positive and negative social associations.

The social cohesion of a choir can be maintained by meeting on-line, especially where the social aspect is particularly foregrounded and facilitated. Indeed, a happy by-product of video conferencing software is that there is potential to see people’s faces who are not normally seen.

seeing others who may be behind you under normal practice conditions and more social interaction [Male; 65–74; facilitator; Live T; Qu 52].

Indeed, even when online music making was abandoned, due to limitations of technology and inability to hear each other and sing together, some found that the social benefits of choir membership could be maintained by shifting the focus of the virtual choir sessions, to foreground social activities rather than musical activities.

We tried singing only once, and it was a disaster because of everyone’s different bandwidths, so we now just use it as a social occasion [Female; 55–64; facilitator; MT; Live T; Qu 39].

However, this chance to maintain social contact is lost where choirs undertake multi-track choir recordings, with many missing the chance to hear others and socialize.

You don’t get the physical interaction of being with everyone else and you can’t of course hear what everyone else is singing at the time of recording [Female; 55–64; Member; Both; Qu 16].

I miss the joining together, the fellowship, the corrections and interruptions of our choir director, and the sociability [Female; 65–74; Member; MT; Qu 19].

Although the virtual choir models differ in their social function, when asked whether they felt their virtual choir provided the same social benefits of in-person choirs, facilitators across both models reported partially or probably not [multi-track: Probably, yes 1.7% (4), Probably not 66% (158), partially 33% (78)]. Live: Probably, yes 2.2% (9), Probably not 47% (189), partially 50% (399).

The sense of no longer being “part of something” was keenly felt in tandem with missing the social aspects of choir membership.

The social aspect is much less online. The feeling of being part of something bigger than myself [Female; 75–84; Member MT; Qu 24].

There is every disadvantage. You can’t hear voices on either side of you. You feel exposed. If you are uncertain of the music you are not helped by being in the virtual choir. You have no real contact with other choir members and, speaking for myself, the experience certainly did not make me feel part of a group [Female; 75–84; Member MT; Qu 24].

In another sense, a common feeling of “being part of something” was shared across the virtual choir models, whether creating solo recordings for an multi-track choir and so being part of a creative “project” or connecting with a choir through a live session, knowing that people were sharing an experience in real-time (although unable to hear each other).

It gives us a real sense of belonging and being part of something [Male; 75–84; Member; MT and Live T; Qu 53].

Indeed, being part of a multi-track recording does in fact offer choir members the chance to feel that they are participating in a performance and that they are contributing toward something that is bigger than the sum of its parts.

Not entirely fulfilling but enjoy hearing/seeing finished performance and makes you feel Part of the choir again [Female; 65–74; Member; MT; Qu 19].

The new socially distanced situation imposed during lockdown also seemed to engender some positive by-products, perhaps due to a collective sense of wanting to ensure that the sociality of choir rehearsals was maintained and communities nurtured.

It is keeping us together as a choir and also gives us time to talk together which we do not normally do [Female; 65–74; facilitator; MT and Live T; Qu 39].

The sense of loss of being able to meet in-person was keenly felt even within positive attitudes toward doing their best with a virtual model, working hard to maintain social links.

I miss meeting in person so much. Online was ok for the first few weeks but then my interest in it waned [Female; 55–64; Member; Live T; Qu 53].

### Musical Elements

The specific musical, and music related, elements of singing as a choir were identified by choir members and facilitators, with some positive by-products of meeting online being highlighted. Often this was mentioned where the choir facilitator had taken extra steps to work creatively within the new situation of running a virtual choir.

Also there is a sense of finding new ways of doing things. And therefore finding new pleasures and new rewards. And it gives so many educational opportunities. And a chance to explore the music [Female; 65–74; Member; MT and Live T; Qu 23].

Some choirs were able to work on new musical material, potentially in a way that they had not done before, where traditional rehearsals focused on preparing a set number of pieces for eventual performance, usually toward the end of a set rehearsal period.

We’ve had a varied program learning new music and techniques rather than rehearsing one or two pieces for a concert [Female; 65–74; Member; Live T; Qu 23].

For others the lack of support from stronger singers in the choir seems to stifle the choir’s collective ability or appetite for learning and investigating new repertoire.

no togetherness feel, no support from stronger singers, lack of learning opportunity or expanding repertoire (sic) [Female: 55–64; member; MT and Live T; Qu 24].

For some the experience of participating in a virtual choir hampered the development of musical/vocal skills, and they were aware that the usual amount of progress, in learning specific pieces, or developing musical skills more generally, was not able to continue online. The lack of performance as an end goal as well as not being positively influenced by other singers around them contributed toward this sense of stultification.

I am more aware how bad I sound, overall we are not making the usual level of progress and we don’t have the incentive of preparing for an actual performance [Female; 55–64; facilitator; MT and Live T; Qu 46].

In addition the absence of choral directors being able to hear individual contributions from choir members (due to technology limitations and everyone having to be muted) meant that the focused and deliberate singers’ skills development could not take place in the usual way.

Developing members (sic) musical ability by targeted feedback is not possible if you can’t hear them. This is a big part of our normal live group rehearsal [Female; 45–54; facilitator; MT and Live T; Qu 46].

For others the virtual choir allowed continuation of some kind, enabling regular singing activity, through which choir members could maintain or further develop musical and vocal skills; often this was further supported by video training materials produced specifically for choir members, or through choir members themselves seeking out existing online resources. Live online choir responses related to music were most often connected to musical learning ability being positively affected, maintaining or improving musical/vocal skills and enabling musical and repertoire growth.

This on-line choir life helps me to continue to sing regularly and improve my technique, thanks to You Tube (sic) sessions, recorded by our Director [Female; 65–74; Member; MT and Live T; Qu 53].

Being aware of individual vocal contributions is unavoidable in virtual choirs, and as a result some members embraced this opportunity to improve their skills, whilst others found themselves unsure of accuracy of notes without a conductor able to provide feedback. This was especially felt for those singing music with multiple parts.

No feedback on your actual part and how you are singing and unable to hear how the whole choir sounds together [Male; 55–64; Member; Live T; Qu 24].

You are forced to identify with your own tunefulness, and not rely on other singers to cover you when you go wrong [Female; 55–64; Member; MT and Live T; Qu 53].

The exposing nature of both virtual choir models, in that you can’t “hide” behind other voices some found enjoyable, but others found inhibiting. Even knowing that no one can hear them sing during live virtual choirs some members reported losing confidence or even stopping participating. Members who were offered an multi-track experience sometimes chose not to participate or submit their recordings because they didn’t like the solo track they had recorded, whilst others relished the opportunity to reflect on their own vocal technique and the chance to re-record tracks until they were happy.

Lost the will to go back. No confidence in singing online with headphones. Online choirs expose my inabilities and the lower standard than I thought of the choirs I’m in [Male; 55–64; Facilitator; None; Qu 53].

No one can hear you, so you can make up new harmonies, sing while other sections are learning, sing as loud or quiet as you want with no one to judge [Female; 45–54; Member; All; Qu 23].

Whilst musical and vocal skill were identified by multi-track members, including some mentions of repertoire growth, the many comments in the context of positive musical experience were related to enjoying and liking the result and being able to listen to the final musical recording as an audience member.

You get familiar with other software, you get to evaluate what you sound and look like singing – such as enunciating words clearly and visually expressing emotions – it definitely increases your confidence and makes you practice as your voice alone is being recorded. The downside is definitely hearing the other voices at rehearsals but this is compensated by hearing the finished audio visual result which is something you don’t get to see or hear, only the audience get that [Female; 65–74; Member; MT; Qu 53].

### Co-creation Through Singing

Whilst difficult to articulate, this theme encapsulates the essence of the experience of in-person choir singing which is unattainable using current virtual choir models. Its absence in virtual choirs is starkly felt by choir members and facilitators, and is often expressed through association with tangible musical elements such as blending and harmony as well as para-musical experiences and associations of the “magic” of in-person choir singing.

The lack of real-time experience of singing together and the loss that comes with it of a shared coordination of music making and social bonding is highlighted by all virtual models. However, the blending of voices through post-production of the solo recordings into a choral sound in the multi- track virtual choir, goes some way toward providing a sense of “in the moment” satisfaction.

When recording my part it felt less enjoyable and far more pressurized. Felt very exposed and was relieved when I saw/heard the final thing and all voices had been blended together [Female; 55–64; Member; All; Qu 19].

It’s nice to hear the blend and feels more rewarding and gratifying than the other choir experience of learning songs over zoom [Female; 35–44; Member; Both; Qu 19].

Others felt that the multi-track results couldn’t re-create the cohesive sound of voices blending together in real-time.

Multi-Track feels a little more like a group of soloist mashed together, rather than a blend of choral voices [Female; 35–44; Member; All; Qu 19].

Choir members describe the experience they are now missing, of being able to blend voices together, multiple voices blending in unison within the sections of the choir, which then with each other in harmony build into a bigger sound, and the social connection and interaction that arise from that.

*At the start of lockdown, I participated in a couple of Zoom online choirs, one from the choir I am a singer in, and one other. I was shocked at how limited and dispiriting this felt, in contrast to being surrounded by 4 part harmony with friends*…. *Now, 17 weeks down the line, I’m able to sing, and listen to glorious music, but am still deeply, profoundly missing the glorious, all-embracing joy of singing with others [Female 35–44; Member; Live; Qu 24].*

We all miss the powerful swell of harmony that is experienced in a room with a large group of singers. This cannot be achieved via a zoom choir and nothing can replace the benefits, sense if achievement and wonder of it [Female;45–54; Facilitator; Live T; Qu 46].

The lack of emotional connection with other choir members was felt by participants in both multi-track and live choirs. For many it was musical cohesion, harmony and blend which contributed to this sense of emotional experience.

in a real choir you feel a physical and emotional connection with the interweaving and blending of part which is totally missing [Female; 45–54; MT; Q59].

The absence of the shared physical, in the moment, experience of music-making was felt not only for the joint action of singing together, but also performing to an audience.

No live performances, although we have had a session on FB Live as part of a virtual festival. MUCH LESS FUN! [Male 55–64; Member; Live T; Qu 24].

[lack of] Audience participation and reaction [Female; 35–44; Member; MT; Qu 19].

When singing together in-person in the same shared physical space all choir members share the same room acoustic, consciously, and subconsciously reacting to and interacting with their own and each other’s sound in the shared acoustic space. Such a key aspect of shared music making in space cannot be replicated in either virtual choir model.

Choirs develop by the members singing together in a real acoustic and adjusting to what each singer hears. This can only be done in “real time” and in person. By removing this person-to-person contact the essential nature of choir music making is changed, and usually degraded [Male; 75–84; facilitator; Live T; Qu 53].

The lack of a physical sense of sharing space together is also felt by members of multi-track and live choirs.

Singing physically with other people is very morale boosting and the social interaction is vital for my wellbeing [Female; 75–84; Member; Live T; Qu 24].

It’s not the same at all – the vibrations and the personal interactions are missing [Female; 65–74; Member; Live T; Qu 21].

Overall these many diverse aspects of singing together that choir members and facilitators felt were overwhelmingly lacking, combine to make up something “other,” intangible, difficult to describe, perhaps even para-normal, that seems to be at the very heart of the choral experience. This is perhaps the essence of choral singing together that the virtual choir experience so starkly exposes.

For me group singing is an immersive experience, being aware of and interacting with folk around, and involves picking up subtle cues by a musical sixth sense that is not present in the clinical, sterile world of virtual attempts to produce a pseudo-choral experience [Female; 65–74; Member; none; Qu 26].

it is not at all fulfilling, whereas singing together in a choir in reality gives a buzz, relaxes you, it is enjoyable [Female; 65–74; Member; All; Qu 24].

When identifying differences between virtual and in-person choirs the loss of the Co-creation through singing theme was prominent for both live and multi-track models. The overwhelming focus on “Co-creation through Singing” when asked about the disadvantages of virtual choirs contrasts the complete absence of the theme when asked to identify advantages. Positive responses to the question of advantages to virtual choirs were contextualized from a perspective of the unique situation of lockdown, with a sense of participants striving to find the positives that make engagement with their virtual choir a valuable experience within the given situation.

## Discussion

The Wellbeing, Social Aspects, Musical Elements, and Co-creation through Singing themes reflect current understanding of the value of group singing as reported for in-person choirs through investigations of the benefits of group singing from social and psychological perspectives (Bailey and Davidson, 2005; Clift et al., 2008; Busch and Gick, 2012; Livesey et al., 2012; Dingle et al., 2013).

The theme of Choir Continuity, sits firmly within the context of Covid-19 pandemic with virtual choirs being utilized as a stop-gap. For the choir member respondents who did not engage in any virtual choir model, the negatives outweigh any positives, however, the uptake of virtual choirs has illustrated first hand that “online groups can fulfill important emotional and utilitarian needs” (Galston, 2000). The continued engagement of many singing “in spite” of the limitations of virtual choirs highlights the sense of loyalty and responsibility felt by members and facilitators alike toward their usual choir communities. Coupled with the strong focus on the importance of social aspects of choirs, (noted both as a positive aspect of virtual models and as a limited element of the experience) supports the concept of social capital and the signifier of fellowship within it (Langston and Barrett, 2008). The strength of social aspects also parallels the weight of this component found in the study of Scandinavian choirs during the Covid-19 pandemic (Theorell et al., 2020). This Likert scale based questionnaire assessed the extent to which choirs were missing seven different aspects of choral activities, chosen through study of previous literature, due to social restrictions. In addition to greater weight to the social component overall, more importance was placed on this by Norwegian compared to Swedish participants, with the latter reporting more importance to the aesthetic and flow aspects. As this paper doesn’t consider virtual alternatives that might have been explored by these choirs, further cross examination isn’t appropriate, although the overall importance of social elements in choral activities is once again supported.

The purpose of many of the virtual choirs to maintain existing in-person choir communities, creates shared physically based virtual communities of social capital “linking the physical and virtual communities to create a new type of community form, that is, a new ‘space’ or ‘place’; where people can interact with their physical (and virtual) neighbors” (Blanchard and Horan, 1998). Where entirely new “choirs” have been formed with success, these have capitalized on the removed geographical boundaries, bringing together communities of common interest and the forming of virtual Communities of Practice (Waldron, 2009).

Maintaining social interaction was achieved differently across the various virtual models, and seemingly with slightly more success in the live virtual choir. Where the live virtual choirs using tele-conferencing software have been unable to sing together and hear each other, due to the limitations of the software and internet speeds, they have often focused their energy on creating valuable social interactions through quiz nights, and spoken conversation. The live streamed model relies on text-based chat for participants to feel this is an interactive rather than passive activity. The multi-track model also relies on forms of social media to create social connections, or a blended model which might include “live” virtual rehearsals in which spoken or text-based chat can also take place.

Although some positive social aspects were identified they were very much within a context of “better than nothing,” and there was no suggestion from multi-track choirs that their social experience within the virtual choir improved, which is contrary to the evidence of multi-tracks eliciting higher levels of social presence than in-person choirs (Fancourt and Steptoe, 2019). This could be due to the different contexts and methods used in the study, as the majority of participants in our survey were engaging in virtual choirs as their only option for participating in any form of “choir.” The reduced loneliness attributed to virtual choirs for individuals during Covid-19, in which the individuals were already in an isolated situation, does compare to the reduction in isolation and forming of social bonds observed through the K-12 virtual choir education project for distance education students (French, 2017), and relates to theories of music reducing loneliness through a role of social surrogacy (Schäfer and Eerola, 2017) which are discussed below.

Whilst the models of virtual choir are strikingly different in their process, they elicit very similar responses associated with the emergent themes. This is understandable considering the connections between their function and the communities they are trying to maintain. There are two differences in the experience reported across the live and multi-track models that stand out within the subthemes: a lesser sense of beneficial social aspects in the multi-track choir, but an indication toward some element of co-creation through musical singing experience via listening to the “choral” sound produced from the solo multi-track recordings. This provides an almost delayed “in the moment” experience, especially with the known context and memory of performing together in-person: listening to music alone may induce a variety of significant endocrine effects (Kreutz et al., 2004) and facilitate relaxation (Krout, 2007). The associations of these recordings with both the Covid-19 situation and the memory of in-person performance is likely to heighten this response. Listening to music has been found to provide a social surrogate, providing a sense of belonging and comfort different to that associated with characters in other forms of social surrogate such as literary or TV characters (Schäfer and Eerola, 2017). The focus on creation of music through singing together, rather than passive listening, makes direct comparison with such literature premature, although social surrogacy may be at play for those finding comfort in the final products of the multi-track choir, similar to the reduction in loneliness observed by Schäfer et al. (2020) for private musical engagement. Whilst most members reported enjoying the final product of the multi-track model, some expressly did not at all and it may not have been an uplifting experience for all. For those who found the results less satisfying and even distressing to listen to, the association may heighten the sense of a lost activity, which aligns with the findings of Kreutz et al. (2004) who observed an increase in negative mood of singers when passively listening within their usual choir rehearsal. The active process of engaging in virtual choirs seems not to have functioned as a social surrogate for many, suggesting that the lost essence of the experience is felt more keenly than the elements that the virtual models can provide. The questionnaire did not ask participants about their musical listening beyond their choir experiences. It is likely that other music listening, especially that not directly associated with their own loss, might still act as a social surrogate for these individuals and would be an interesting area of further study.

Common to both live and multi-track models was the essence of the experience meaning that you are singing alone, with regular comparisons made to karaoke singing, especially for multi-track choirs, and this was overwhelmingly identified as a negative element of the experience. The acoustic complexities involved in group singing far exceed issues of tuning and harmony, with multiple dynamically adjustable acoustic features being created and altered by the voices which are adapting to each other through complex relationships that result in a “blended” choral sound (Ternström, 2003). Some found the process of singing alone upsetting, perhaps in light of it reminding them about the experience they want but can’t have. Others reported enjoyment, uplifting mood, and general positive effects to their wellbeing that might be expected with the triggered endocrine response associated with solo singing (Schladt et al., 2017).

The exposed nature of solo singing in virtual choirs elicited diverse reactions from choir members, with some taking an opportunity to reflect and improve on their vocal and musical skills whilst others were put off the experience entirely and their confidence negatively impacted. For the latter group virtual choirs may shift the “group process” identified in Bailey and Davidson’s theory of positive effects of group singing towards “social support” (Bailey and Davidson, 2005). In their theory “Social support” was associated only with the group they termed marginalized, who were homeless or living in impoverished communities, whilst “safe environment to experience voice” was associated with the middle-class choristers (Bailey and Davidson, 2005). Whilst personal circumstance and socio-economic background were not collected for respondents in this survey, it is unlikely that it was completed by marginalized individuals in these terms. It would be interesting to explore groups from different socio-economic backgrounds engaging with virtual choirs, to see if there would be less negative association with the vocal exposure of lone singing, as the marginalized individuals, unlike middle-class choristers, were not inhibited by prevalent social expectations of musicianship (Bailey and Davidson, 2005).

Unanimous across all respondents was a frustration and sadness at not being able to sing together, in-person. The difficulties found in trying to describe what it is about singing together that respondents consider special are also apparent. Many articulate this difficulty but very few respondents try to fully describe the unique experience of singing together in a room very precisely. However, there are many descriptions of “parts” of the “magic” of the shared experience of singing together, reflecting the observations of Specker who found “Participant reflections on the shared sonic experience were fluid and complex, often encompassing multiple categories and concepts … The process of vocalizing sound together brought about implicit feelings of mutual ‘tuning-in’ and connection, creating feelings of collective unity in a specifically sonic manner” (Specker, 2017, p.115). The importance of embodied, physical, sonic, shared experience, which is lacking in all of the virtual choir models, has been indicated to have significant impact on indicators of stress and arousal, as well as inducing social flow (Keeler et al., 2015).

The shared physical and sensory experience of making music together that strengthens social bonding (Tarr et al., 2014), once removed in the virtual models, became starkly apparent. Interpersonal coordination does not take place in a virtual choir, and therefore any potential for the experience of entrainment (Clayton, 2012), or mutual flow states (Keeler et al., 2015) is lost.

Links between musical experience and social connections are strong. The emphasis on the absence of the complex real-time adaptations of singers as they adjust their intonation and “blend” their voices to one-another creating “cohesion” supports the commonly held view that there is something special about the shared creation of a sonic experience as opposed to other group activities (Stewart and Lonsdale, 2016) and that the embodied act of mutual sonic creation is not easily separated from the social connectedness arising from choral singing (Specker, 2017).

Explored within the framework of social cure, the greater the social identity formed within a group activity the greater the wellbeing benefits that have been observed (Williams et al., 2019). Within this framework, group singing, whilst not found to have any more impact than a creative writing group, has contributed to the evidence supporting the role of arts-based therapies for adults with chronic mental health problems (Williams et al., 2019) engendering a sense of belonging, support, self-efficacy, purpose, and positive emotions (Williams et al., 2020). Also utilizing the social cure approach, Forbes (2020) observed new and valued social identity formed through a group singing program for Parkinson’s spouses, as a “psychological resource in the form of connection, meaning, support, and agency” (pp.7) and helped them manage the impact of the diminishing effects of the disease. Similarly, a randomized control trial of a group singing program for older adults in a retirement home found positive social and cognitive effects, although with no significant effect on wellbeing (Dingle et al., 2020).

This study did not directly assess mental or physical health, and with no baseline measures relies on the self reported change felt between in-person and virtual choir experiences. However, a dominant message of participants was the strong sense of social identity associated with their usual in-person choir situation, both in terms of trying to hold on to it through the virtual models and feeling its loss. That the virtual models do not provide a consistent reported sense of self identity across participants, with those not taking part often citing increased loneliness and lack of community felt with the virtual models, suggests that different aspects of the activity contribute to the social identity and sense of community for individuals.

There is evidence of a strong understanding amongst conductors of their role in developing social agency and identity of a choir (Durrant, 2005) and a keen sense of responsibility in maintaining a sense of community and the success they take pride in having nurtured (e.g., Bell, 2008). Certainly members and facilitators alike sought to maintain the strong sense of community formed through their in-person choirs, and the role of the conductor or leader of the virtual choir experience was highlighted as extremely important to the positive engagement of members. Conductors were required to adapt, quickly, to an often entirely new situation and commit extra time (usually unpaid), extensive learning of new skills, and immense energy into the preparation and running of sessions, which was greatly appreciated by members.

Considering the different virtual choir models that have been employed during Covid-19 alongside their practical and technical limitations and the reported value of the experience by members and facilitators, a blended model incorporating both live and multi-track would be the ideal approach in maximizing the potential benefits of virtual choirs. The social aspects of interaction and spoken conversation can best be delivered over video conferencing software, which also allows musical “practice” whereby, although hearing only singing along to perhaps a lone reference voice due to the necessity to mute, there is a sense of collective action in knowing everyone else is singing too. In order to feel a musical reward, a multi-tracked virtual choir is the only model that can provide a somewhat “choral” aesthetic and, although through listening rather than singing, a musical reward and a delayed sense of shared co-creation of music. Unfortunately, the time resource needed to run a blended model for virtual choirs make it unlikely to be a feasible medium to long-term solution for choirs. Virtual choirs are often running on good-will and, whilst this study highlights the importance of such experiences and their role in supporting choir members during the pandemic, it really pushes to the fore the centrality of the embodied process of singing together in the value of group singing as a beneficial social activity.

## Limitations and Future Work

There are a number of limitations of the present study, which may affect the context and interpretation of the results and thematic analysis. Whilst effort was made to distribute the survey beyond academic and local networks, the demographic of respondents was self-selecting to people who have access to the internet and the technology and skill to respond to an online survey. Due to this it is likely that, whilst we were keen to consider how choirs had dealt with the pandemic (including those who did not pursue a virtual model), there is a disproportionate sample biased toward choirs and choir members who have engaged with a virtual choir. The age and gender distributions of the sample are also skewed toward older female participants. Whilst this may reflect the general engagement of group singing in the United Kingdom, the high proportion of female respondents may also be indicative of differences in the perceived value in the experience (Vaag et al., 2014). It is possible that some differences may emerge from younger, more tech savvy populations, although unlikely in terms of the limitations of the technology, which can’t be easily removed. We will also have captured the population who are very passionate about choir singing and therefore likely to extol its benefits. In future it would be interesting to consider the efficacy of virtual choirs for “non-participants,” who are reluctant to join a choir, as the differences in participation might allow a valuable route in for engagement with choir singing (Whidden, 2010).

A cross-sectional study of this kind cannot by its nature provide in depth, longitudinal exploration of the experience of individuals involved in virtual choirs, and field study of smaller samples to triangulate with the larger dataset provided by the survey data would be highly valuable. In particular, it will be of interest to consider the longer-term engagement of choirs with virtual models once the restrictions brought about due to Covid-19 are removed and as the technology improves.

The theme of Participation Practicalities is highly specific to Covid-19, especially with the strong focus on technology which dominates the logistical barriers to virtual choir participation. The positives identified within this theme, however, in particular the lack of travel involved, highlight the potential opportunities that virtual choir models might provide such as increased accessibility of choir communities across different populations. An existing immersive virtual reality choir experience, which relies on pre-recorded group singing material, is already being used in residential care homes to provide access to group singing experiences with positive results (Daffern et al., 2019). In order for the experience to be truly one of “group singing,” however, the technology needs to be developed to allow at least some of the elements of “real-time” coordinated actions of music-making to be felt. The reported experience of the practical opportunities of the virtual choirs during Covid-19 align with the findings of telehealth initiatives for the delivery of music therapy, whereby online delivery provides continuity, familiar home environments and access for geographically remote individuals or those physically unable to attend (e.g., Vaudreuil et al., 2020). Utilizing virtual reality Tamplin et al. (2020) found similar benefits to real-time therapeutic singing interventions for people with spinal cord injury, with participants reporting reduced inhibitions about singing in front of people and good levels of immersion. A virtual reality experience was created using the low-latency audio software JackTrip in order to overcome the barrier of latency using tele-conferencing software such as Skype.

Several bespoke Networked Music Performance (NMP) softwares exist which address issues of latency and seek to enable realistic group music performance, with most success for very small ensembles (Rottondi et al., 2016). However, implementation requires expert levels of technical expertise and they often rely on academic networks rather than being suitable for home internet connections. Only one participant mentioned engagement with such software (Jamulus). As internet speeds increase alongside computational speeds, this technology needs to become easier to use and needs developing further to function within the necessary latency targets required for singing together in real-time over home networks. Such technological improvements would also create opportunities for further development and uptake of the online delivery of music education to remote communities (King et al., 2019), and exploration of e-collaboration and theoretical frameworks for music learning in digitally deterritorialized collaborative spaces (Cremata and Powell, 2017). With immersive audio techniques rapidly advancing there is also the potential to produce a shared acoustic environment within a real-time live virtual choir, whereby participants could hear themselves and other choir members in a shared virtual space in their given position with the group (Cairns et al., 2020). If members could sing *together*, to tune with each other and coordinate their vocal outputs into a shared sonic experience, engagement with virtual choirs would almost certainly increase with more positive benefits observed. Such developments in the technology would also present opportunities to systematically investigate the impact of sharing a physical and acoustic space on the embodied experience of group singing.

## Conclusion

Virtual choirs provided a lifeline to many singers during the Covid-19 lockdown in the United Kingdom. The possibilities of technology have illustrated the potential access that it could afford to group singing experiences in more normal times. However, the limitations imposed by the current virtual choir models which cannot provide the shared experience of singing together, have highlighted the recognition of the importance of in-person group singing for perceived wellbeing, with the connectedness and social capital formed in the process of singing together being central to the benefit of this experience. The study of virtual choirs has the potential to improve our understanding of the complex multidimensional relationships contributing to the value of the experience of singing together, in a shared space. Future developments in technologies for networked music performance need to capture some of the essence of group singing as a shared experience, likely to be improved through implementation of immersive audio techniques within narrow latency limits. With such systems in development and internet speeds continuing to increase, a *virtual* choir has potential to become a reality soon, but only if the technology is developed hand-in-hand with a better understanding, afforded by this and similar research, of what makes singing together so unique, highly valued and ultimately “magic.”

## Data Availability Statement

The raw data supporting the conclusions of this article will be made available by the authors, without undue reservation.

## Ethics Statement

The studies involving human participants were reviewed and approved by the University of York Physical Sciences Ethics Committee. The patients/participants provided their written informed consent to participate in this study.

## Author Contributions

HD: funding acquisition, conception, distribution, design, coding, analysis, writing, and editing. KB: design contribution, distribution, coding, analysis contribution, and editing. JB: design contribution, coding contribution, analysis contribution, and writing contribution editing. All authors contributed to the article and approved the submitted version.

## Conflict of Interest

The authors declare that the research was conducted in the absence of any commercial or financial relationships that could be construed as a potential conflict of interest.
