# Supplementary material for: Singing Together, Yet Apart: The Experience of UK Choir Members and Facilitators During the Covid-19 Pandemic
Source: Front Psychol. 2021 Feb 18;12:624474. doi: 10.3389/fpsyg.2021.624474 (PMC7930073; doi:10.3389/fpsyg.2021.624474)
Supplement: Supplementary file 1 [file Table_1.DOCX]

Appendix 1: Inventory of questions asked in the survey of UK choirs during the Covid-19 pandemic of 2020

Demographic / General Questions

1 How old are you?

*Multiple Choice: 18 – 24, 25 – 34, 35 – 44, 45 – 54, 55 – 64, 65 – 74, 75 – 84, 85 or older*

2 What gender do you identify with?

*Multiple Choice: Female, Male, Non-Binary, Other, Prefer not to say.*

3 Before lockdown did consider yourself to have a disability, learning difficulty, long term health condition

*Multiple Choice: Yes, No, Prefer not to say*

4 During lockdown has your wellbeing, physical health and mental health
*Multiple Choice: Improved, Stayed the Same, Declined*

5 How many choirs do you participate in as either a facilitator (e.g. Director / Conductor / Organiser), accompanist or singer?

6 What best describes your choir(s)? (Select as many as apply)
*Multiple Choice: Church Choir, Chamber Choir, Community Choir, Children's Choir, Therapeutic Church Choir, Barbershop Choir, Large Chorus Choir, Solo Voice Ensemble, Gospel Choir, Bach Choir, Jazz Choir, Pop Choir, Rock Choir, Show Choir, Folk Choir, Mixed Choir, Workplace Choir, Other, if so please state:*

7 Are you a choir facilitator (e.g. Director / Conductor / Organiser) or a choir member? (if both please tick facilitator)

*Multiple choice: Choir Facilitator, Choir Member*

Virtual Choir Questions:

8 Did (any of) your choir(s) exist before the COVID-19 pandemic?

*Multiple Choice: Yes, No*

9 Where in the UK is your choir(s) located?

10 How long have you been a member of the choir(s)? (select all that apply)

*Multiple choice: Under 6 months, 6 months to a year), 1-2 Years, 3-4 years, 5 Years+ .*

11 How often do your choir(s) meet? (select all that apply)

*Multiple choice: 4 or more times a week, 2 to 3 times a week, Once a week, Once a Month.*

12 How often do you normally attend? (select all that apply)

*Multiple choice: I attend all practices, I only attend some practices, I rarely attend practices.*

13 How many people usually attend? (select all that apply)

*Multiple choice: 1-20, 21-40, 41-60, 61-80, 81-99, 100+ .*

14 How many of your choir(s) have continued in any form since the COVID-19 induced lockdown?

*Multiple choice: None , 1 , 2 , 3 , 4, 5+*

15 Have you joined any choir(s) that began during lockdown?

*Multiple Choice: Yes , No .*

16 What type of virtual choir have you been taking part in? (select all that apply)

*Multiple Choice: Multi-Track ((Making your own video or audio recordings at home so that they can be edited together), Live (via an internet call on platforms such as Zoom or Skype), Live (via an internet call on platforms such as Zoom or Skype)*

17 How many Multi-Track performances opportunities have you taken part in? *Multiple Choice: I have taken all opportunities, I have taken some opportunities, I have rarely take the opportunity, I have not taken the opportunity*

18 Did you enjoy watching the final version once it was all edited together?

*Multiple Choice: Yes, No, Not as much as I expected to, More than I expected to*

19 How do you feel the Multi-Track Choir experience differs to the in-person choir experience? If you haven't taken part, why not?

20 How many Live online Sessions have you been attending?

*Multiple Choice: I Have attended all online session, I only attended some online sessions, I rarely attend online sessions, I haven't attended any online sessions.*

21 How do you feel the Live Online Choir experience differs to the in-person choir experience? If you haven't taken part, why not?

22 Is there anything you would like to change about the way that your Live online choir functions?

23 Are there any advantages to participating in a virtual choir? Please explain your response:

24 Are there any disadvantages to participating in a virtual choir? Please explain you response

25 Have you been asked for additional donations to maintain the choir during COVID-19?

26 Why haven't you attended any online sessions?

Facilitator specific questions

27 How often do you rehearse?

*Multiple Choice: 4 or more times a week, 2 to 3 times a week, Once a week, Once a Month.*

28 How long have you been a facilitator for the choir?

*Multiple Choice: Under 6 months , 6 months to a year, 1 to 2 years, 3 to 4 years, 5 years or more.*

29 How is your choir typically funded? (select as many as apply)

*Multiple choice: Donations, Grants, Profit from shows, Membership, Church choir (5), other if so please specif.*

30 Have you moved to facilitating your choir(s) online?

*Multiple Choice: Yes, No.*

31 How did you find organising a Multi-Track Choir

32 Did you edit the multi-track choir recordings yourself? If so, how did you find it?

33 How have you distributed the final product? (e.g. Facebook, Instagram, Youtube, Email etc...)

34 What is it like to run a multi-track choir compared to an in-person choir?

35 Do you feel that the Multi-Track Choir(s) achieve the same social benefits as attending a choir in person?

*Multiple Choice: Probably yes, Probably not , Particularly.*

36 What online platform has your choir(s) been using for your Live Online Choir(s)? (E.g.Zoom, Facebook, Youtube.

37 How often do you run an online session as a group? *Multiple Choice: 4 or more times a week, 2 to 3 times a week, Once a week, Once a Month.*

38 Has Participation of the Live Online Choir remained at a steady level since starting? *Multiple Choice: Yes, No, it has lowered, No, it has increased.*

39 What is it like to run a choir Live online compared to an in-person choir?

40 Do you feel that Live online choir(s) achieve the same social benefits as attending a choir in person? *Yes, No , Partially .*

41 What type of repertoire have you found works best virtually?

42 How soon after the lockdown began on the 23rd March 2020 were you able to setup the Virtual Choir?

*Multiple Choice: Under a week , 1-2 weeks, 3 weeks - 1 month , 1-1.5 months , 2 months +.*

43 Where did the idea for the Virtual Choir Come from?

*Multiple Choice: Thought of it yourself , Members of the choir suggested it , Inspired by other groups online.*

44 Why did you want to continue your choir virtually?

45 Have there been advantages to running a Virtual Choir? If so please state below:

46 What have the challenges been with running Virtual Choir? These can be musical, technical, social or technology-based.

47 What have you learnt from running the Virtual Choir?

48 How would you rate the general feedback you have received from your online choir's participants?

*Multiple Choice: Only poor feedback, Mainly poor feedback, mixed feedback , Mainly good feedback, only good feedback.*

49 Has there been a financial impact on your choir(s) as a result of COVID-19? *Multiple Choice: Yes, No.*

50 Have you had to apply for additional funding to keep the choir(s) running?

*Multiple Choice: Yes, No.*

51 Have you asked members for additional donations to maintain the choir(s)? *Multiple Choice: Yes , No.*

52 Are there any advantages to participating in a choir online for members?

Final Question

53 Is there anything else you would like to tell us about your choir experiences during the Covid-19 Pandemic?
