# Supplementary material for: Singing Together, Yet Apart: The Experience of UK Choir Members and Facilitators During the Covid-19 Pandemic
Source: Front Psychol. 2021 Feb 18;12:624474. doi: 10.3389/fpsyg.2021.624474 (PMC7930073; doi:10.3389/fpsyg.2021.624474)

Appendix 2: Flowchart of the question structure for the survey of UK choirs during the Covid-19 pandemic of 2020


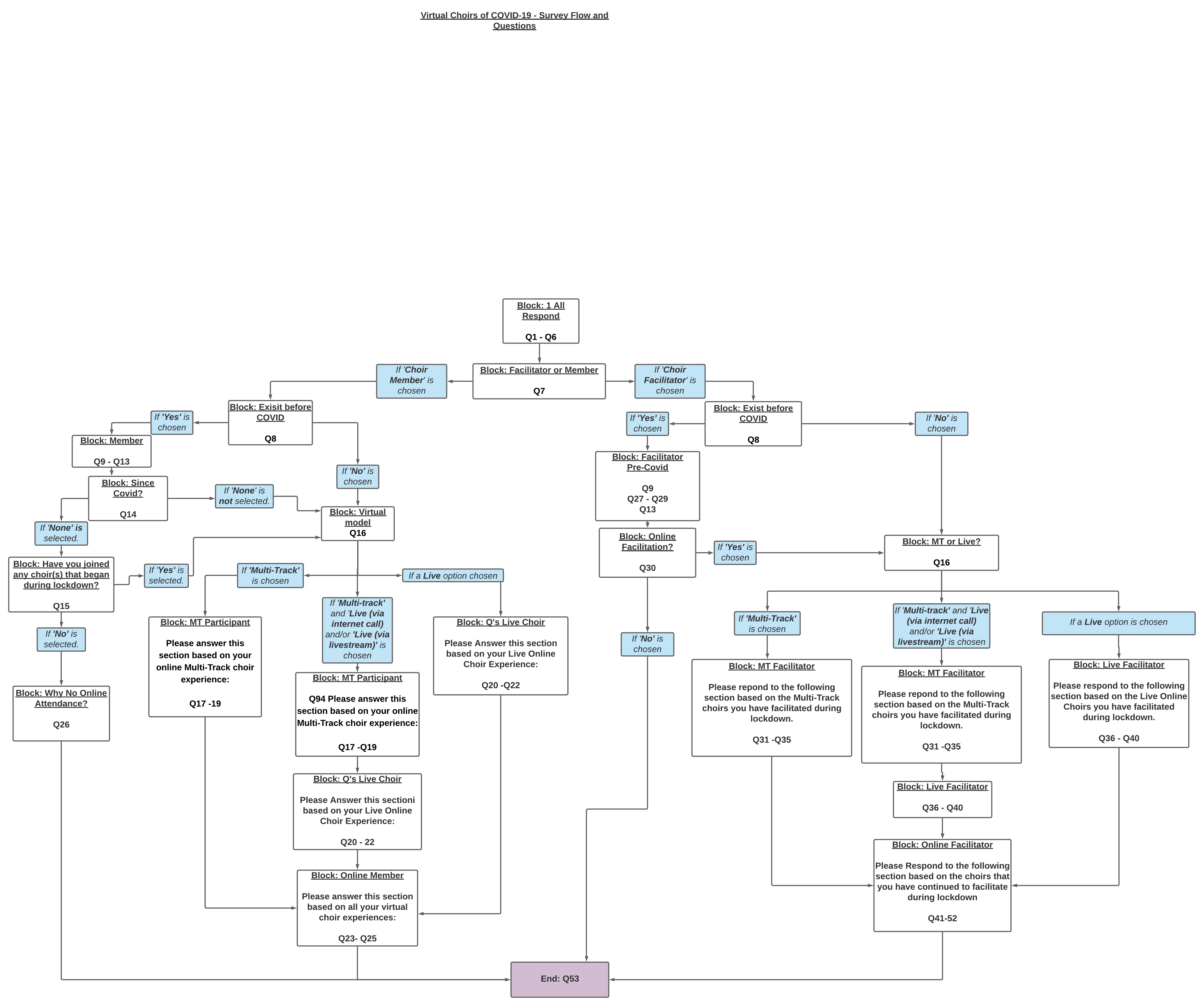

Supplement: Supplementary file 2 [file Table_2.DOCX]
