# Supplementary material for: Singing Together, Yet Apart: The Experience of UK Choir Members and Facilitators During the Covid-19 Pandemic
Source: Front Psychol. 2021 Feb 18;12:624474. doi: 10.3389/fpsyg.2021.624474 (PMC7930073; doi:10.3389/fpsyg.2021.624474)
Supplement: Supplementary file 3 [file Table_3.DOCX]

Appendix 3: Theming Coding Tree generated from responses to the survey of UK choirs during the Covid-19 pandemic 2020.

| **Theme** | **Subtheme** | **Labels** |
| --- | --- | --- |
| **Participation Practicalities** | Hardware/Software | Technical Limitations  Access to technology  Internet not good enough  Internet connection  Latency / Real time  Internet Connection / Latency  Technically Impossible / Limited  Technical Problems  Blue light / sore eyes  Too much screen time  A Lot of Technology  No latency / hardware  Stronger internet connection  Limited tech  Tech allowed choirs to continue  Already at limit of what tech can do |
|  | Skills | Technical Skills  Lack of Technical Understanding  Tech too Complex  No [Expertise] with [experience] tech  Video Editing / Syncing Hard  Hard to Edit  Time /Syncing Problems  Didn’t Like editing  Expert used  Sound Quality  Sound Quality Bad  Pitch / levels issues  Time / syncing problems |
|  | Environment | No space at home  Disruptive Home Environment  Background noise  Interference / background noise  Inconvenient / Work/life clash  Childcare is Easier  No Travel  No Weather Impact  Not Out in Evening  Flexibility of Rehearsal  Attendance Changes  Singing from home |
|  | Access and Inclusion | Excludes Members  Lack of engagement  Hard to engage members  Accessibility  Shielders  (tech)Enabled Participation  Inclusivity  International Connection  Including parent involvement  Inclusive  International  Connect with new People / Choirs |
|  | Effort | Time consuming  Time / effort consuming  Not worth the effort  More preparation  Difficult to run  Hard work  More organization  Challenge  Learning Curve  Easier with Practice  Positive change over time  Easy  Easier  Less effort  Less organisation  Less time consuming  Audio Editing Easy |
|  | Cost | Cost  Expensive  reduced /no cost  Lower cost |
| **Choir continuity** | Better than nothing | Ok  Better than nothing  Different to in-person/real  Return to in person choirs  Mixed View  Not the same  Interesting  Strange |
|  | A Stop Gap | Ok as a stop gap  Not a long-term solution |
|  | Economic | General Income  Economic Concerns  Personal Income  More Funding  Business |
|  | Responsibility and loyalty | Social responsibility  Commitment Responsibility  Satisfying Requests of the choir  Pastoral Communication  Gain membership  Maintain Choir Interest  Sustain Choir Membership |
|  | Motivation and Engagement | Hard to motivate  No motivation |
| **Wellbeing** | Wellbeing | Positive/Improved Wellbeing  Positive effect on wellbeing  Improved wellbeing  Negative effect on Wellbeing  Tiring  No/Less Wellbeing |
|  | Sense of Purpose | To Give Individual Purpose  Religious |
|  | Stress | Stressful  Burden on editor  Burden on facilitator  Stressful for facilitator  Stressful for singers |
|  | Loneliness | Loneliness  Part of something  Reducing loneliness  Lonely |
| **Social Aspects** | Social contact | Social aspect positive  Improved social aspect  Social Aspect  Social Communication  Connect with new people / choirs  Social Aspect Missing  Missing Social Aspect  Missing interaction |
|  | Being part of something | Impersonal  Not part of something  Sense of participation missing |
|  | Community | Community atmosphere  Community Spirit  Supporting  Lacking community |
|  | Human Contact | Lacking physical contact |
| **Musical Elements** | Musical Growth | Musical Growth  Repertoire Growth  Musical Growth  Maintain Musical Standard(s)  Lacks musical growth  Unable to develop (musical/vocal) skills  Love for music  Importance of music |
|  | Vocal Skill | Vocal technique  Musical / vocal skill  More individual practice  Maintain Practice / Singing  Maintain Practice  Individual vocal technique exposed |
|  | Musical Skill | Musical skills focus |
|  | Accuracy | Accuracy improvement  Accuracy improvement  Accuracy concern  Uncomfortable  Felt less confident singing  Part singing difficulties  More responsibility for singers |
|  | Musical Confidence | Felt More Confident Singing  Lacks confidence  Need confidence |
|  | Learning / Teaching | Learning Ability Positively affected  Learning Opportunity  Easier to teach  Teaching members  Understanding choir ability  Sing different parts  One-to-ones  To try something new  Creative |
| **Co-creation through singing** | In the Moment | No cohesion  Lacks simultaneous  Missing acoustics  Musicality impaired  Less musically rewarding  Not musically rewarding  Lack of conductor  Lack of music making |
|  | Magic | Magic  Sixth sense  More energy needed |
|  | Musical Cohesion | Not singing together  Singing alone  Unable to hear each other  Unable to hear / sing with each other  Can’t sing together  Sing along / karaoke  Missing singing  Music Making  Missing harmonies  Can’t sing together  Lacking vocal support from other members  Real-time feedback missing  Exposed while taking part |
|  | Physicality | Physicality missing  Not being together |
|  | Emotional connection | Lacks emotion  Uplifting  More emotional  Joy of Singing |
|  | Audience | Missing performance  Audience present  No audience  Listening from audience perspective |
