# Supplementary material for: Singing Together, Yet Apart: The Experience of UK Choir Members and Facilitators During the Covid-19 Pandemic
Source: Front Psychol. 2021 Feb 18;12:624474. doi: 10.3389/fpsyg.2021.624474 (PMC7930073; doi:10.3389/fpsyg.2021.624474)
Supplement: Supplementary file 4 [file Table_4.docx]

Appendix 4: Themes and subthemes with Illustrative responses from the survey of UK choirs during the Covid-19 pandemic. Relevant information is provided in brackets after each response Gender; Age; Role; Virtual model; Question. MT = multi-track; Live T = Live tele-conferencing; Live S = Live streamed.

| **Theme** | **Subtheme** | **Illustrative responses** |
| --- | --- | --- |
| **Participation Practicalities** | Hardware / Software | *Fairly straightforward but many people have struggled with the technology which hindered participation [Male; 65-74; Facilitator; All; Qu 31]*  *Initially the technical setup; broadband speed, needing Ethernet cable, having everything in the right place and at the right height [Female; 35-44; Facilitator; Live T; Qu 46]*  *A few singers don't have access to IT so we've had to rely on dial-in connections, which restricts what I can do in rehearsals without excluding those members [Male; 45-54; Facilitator; All; Qu 46]*    *Social - You cannot replace real human warmth, hugs and social connection. Miscommunications are more prevalent in online situations because it's not a real conversation with all the nuances and subtleties of full communication - Choir WhatsApp groups can get into trouble with this! Technological - Also you cannot sing together in time on Zoom so no one can hear each other nor can I hear them except for very slowed down mad singing - so it's impossible for me to know how everyone is really doing. Access - getting online is not accessible to everyone, and can be very confronting to those who are older/ technophobic.* *Whilst we have managed to learn songs - It's just not choir - it's more like a sing-a-long with me or with audios. For some groups this is ok but for more ambitious groups who are all about the harmonies its vastly dissatisfying.It's hugely difficult to know where people actually doing and how they are feeling - so much feedback is picked up without words from being in a room together. Even though it helps us stay connected it also sometimes emphasises isolation, especially having to turn on the mute button and then sing without any feedback to people who are all also in their own boxes. [Female; 35-44; Facilitator; Live T; Qu 46]*  *Internet connection in this rural part of Suffolk/Essex has prevented us from doing choirs online - due to the varied delays. [Female; 55-64; Facilitator; None; Qu 53]*  *We tried sin[g]ing only once, and it was a disaster because of everyone's different bandwidths, so we now just use it as a social occasion [ Female; 55-64; Facilitator; MT and Live T; Qu 39]*  *Would love to be able to sing together and hear everyone. I understand that there is a slight delay and so if we’re not all muted on Zoom it would sound cacophonous. [Female; 55-64; Member; Live T; Qu22]* |
|  | Skills | *I do not have sufficient technology or know how [Male; 75-84; Member; MT & Live T; Qu 19]*  *Availability of suitable technology and skills needed [Female; 65-74; Member; Live T; Qu22]*  *[choir name] Choir has effectively closed since March 2020. Many of our members are elderly and less capable with technology; video conferencing has proven impractical for choral singing. We keenly feel its absence! [Male; 45-54; Facilitator; None; Qu 53]*  *See above- i t is not at all fulfilling, whereas singing together in a choir in reality gives a buzz, relaxes you, it is enjoyable. And that is just the choir bit: the additional fight with unreliable technology just makes an unsatisfactory experience a really stressful one. [Female; 65-74; Member; All; Qu 24]*  *In my particular case I have been editing the Multi-Tracking and so have learnt some new skills and how different people sing. Producing a finished result that is worthy of public performance from all of the individual contributions is satisfying. [Male; 65-74; Member; MT; Qu 53]* |
|  | Environment | *I have recently had an operation and I was still able to attend practice as I didn't have to travel. We have recorded some lovely songs to share on Facebook and YouTube . [Female; 45-54; Member; Live; Qu 24]*  *I miss the support of voices around me which give me confidence to sing out. It's difficult to sing at full strength in case I disturb the neighbours! I miss the social side - particularly our smaller choir of 12 which has been meeting in someone's house for over 10 years - we always have a glass of wine and a chat at the end of the session. [Female; 65-64; Member; MT; Qu 24]*  *Not easy to sing fully in a constrained home environment -- disturbance to others, noises off and disruption. Not easy to sing fully without other people singing around one: singing to a full-choir backing tape can help with this, but singing to a basic backing tape with just a piano playing (eg) leaves a lot of the musical space to be filled in. [Female; 45-54; Member;MT; Qu 24]*  *Time and financial advantages .No travel time or expenses. No hire of hall fees. More flexible- can sit with a cup of tea etc. Can join in to suit - not committed to the whole session if something else impending but able to take part in some of it rather than having to miss session. Can virtually meet new people from anywhere in the world. Increase contacts. . (sic) [Female; 65-74; Facilitator; Live S and Live T; Qu 52]* |
|  | Access and Inclusion | *Having members reconnect from far away and bringing choirs from different locations together. [Female; 45-54; Facilitator; Live T; Qu 45]*  *Easier for many, as our Singers are Seniors and prefer not to travel, so attendance has been more consistent than meeting face to face, even before the pandemic. [Female; 65-64; Facilitator; All; Qu 39]*  *I loved it- it gave me a chance to participate! [Female; 35-44; Member; MT and Live S; Qu 19]*  *It differs because of the limited number of people gathered. It differs because of the time frame of course you can only speak to another person when unmuted which is rare. There is one experience which if it wasn’t for coved 19 I would never have had the opportunity to do, and that is to sing with Opera North Choral. Amazing. [Female; 65-74; Member; Live S and Live T; Qu 21]*  *seeing everyone. Vocal warmups - it is really noticeable if you don’t sing for any length of time. It has presented different challenges from normal that I have enjoyed. The experience of other choirs from normal (eg Eric Whitacre) has been wonderful - I have learned some much from people I would not normally be involved with. [Male; 54-54; Facilitator; All; Qu 52]*  *I did not attend regularly as I was still working. I know for those who did, it united them. They formed online friendships and can't wait to meet each other properly in the future. For one particular friend it provided a lifeline when she was shielding and all her other forms of social contact had [Female; 55-64; Member; Live T; Qu 53]* |
|  | Effort | *As a singer, the experience has been interesting and fun to do, with the added anticipation of the final recordings. But, of course, we can’t wait to be able to get back to singing together and performing to live audiences. Those involved in the sound and video editing spent many hours on their efforts, possibly facilitated by being in lockdown and self isolation situations. [Female; 55-64; Facilitator; All; Qu 34]*  *took a long time to put the video tracks together using editing software [Male; 35-44; Facilitator;Mt and Live T; Qu 32]*  *technical, cost of equipment, it takes more time to organise, set-up and strike, socially poor, energy required is more, financial benefits are less. [Female; 45-54; Facilitator; Live T;Qu 46]*  *Learning new tech, keeping it fresh with new ideas, prep time has increased by an unimaginable amount. [Female; 35-44; Facilitator; All; Qu 46]*  *Although recording your part alone at home feels odd at first, the more you participate in, the easier it becomes. Online social groups help keep give the lone singer a sense of community. However, the experience can never, ever replace singing live with others! As a stop gap it offers an opportunity to learn new works and keep one’s vocal chords exercised. [Female; 65-74; Member; Multi-track; Qu 19]* |
|  | Cost | *less paperwork required and cost of hire of a hall not required [Female; 45-54; facilitator; Live S; Qu 45]*  *The online learning tracks have been very popular and form a valuable resource for the future. Some choir members said that they have taken part when they might not have wanted to drive to choir rehearsals. Saved in rehearsal venue costs [Female; 55-64; Facilitator; MT and Live T: Qu 45]*  *Challenging but immensely rewarding.We are about to release our second recording in support of a local charity and the confidence of members has improved enormously around doing this sort of thing. It was hard to convince them to do it on board but the results were worth it. It has been extremely time consuming but it has been a great exercise in learning about individual members and their voices. It has also been an extremely positive force for the choir, with our first attempt raising over £3000 for charity.[Male; 25-34; Facilitator; Live S and Live T; Qu 31]* |
| **Choir Continuity** | Better than nothing | *Nothing but nothing replaces the experience of live choral singing. Virtual and Zoom Music is a lot better than nothing, but cannot replace the real thing. Even though it might have to. [Male; 65-74; Facilitator; None; Qu53]*  *Better than nothing but vastly inferior to live experience [Female; 65-74; Member; Live T; Q21]*  *Social thing I suppose - but pretty good substitute [Female; 65-74; Member; MT and Live S; Qu 24]*  *It is a pale substitute for the real thing but nonetheless important for morale, continuity and wellbeing [Female; 65-74; Member;All; Qu21]*  *Not really - it’s better than not meeting at all. [Female; 65-74; Member; MT and Live T; Qu 24]*  *I miss singing so much. Music rehearsals are like a kind of meditation - they push all life’s daily stresses away and rejuvenate you. I hadn’t realised what a big part collective music played in maintaining my balance/wellbeing until it was removed. You simply cannot get the full experience by singing into your computer against a click track. Although it is better than nothing. [Female; 35-44; Member; MT; Qu 53]* |
|  | A stop gap | *I am pleased to have taken part in my choir leader's virtual choir so that I can keep in touch with her and other choir members. However, I am so looking forward to being able to meet normally. [Female; 65-74; Member; Live ; Qu 53]*  *Although recording your part alone at home feels odd at first, the more you participate in, the easier it becomes. Online social groups help keep give the lone singer a sense of community. However, the experience can never, ever replace singing live with others! As a stop gap it offers an opportunity to learn new works and keep one’s vocal chords exercised. [Female; 65-74; Member; MT; Qu 19]* |
|  | Economic | *I chair the chamber choir I sing in. The committee and I have worked hard to support our inspirational MD in creating a programme which has engaged a majority of members to some extent. We worry about how sustainable this is in the long term however. In particular, members of working age who are spending long hours daily on video meeting platforms are becoming less regular attendees at choir sessions. The social aspect of both choirs is highly valued and its loss will have serious consequences. Long term, our financial viability is a concern. [Female; 55-64; Mt and Live T; Qu 53]*  *The two online choirs I have joined during the pandemic (neither of which charge fees) are fantastic. I can’t thank the people who have given up their time and money to put these on for people around the world. I am equally sad that due to the nature of it, my large chorus community choir that I sang with before the pandemic has not been able to continue by moving online - it would just not be feasible for either our director or the vast majority of singers. While we are lucky in that we have managed to retain a healthy bank balance and therefore will still be there when the government decides to allow us to meet in person again (the differentiation between professional and amateur groups in their guidance seems discriminatory and ludicrous to be honest - does being an amateur and unpaid choir make your members somehow more vulnerable to COVID-19 then a paid professional choir?), however I am concerned for the numerous smaller community choirs around the country who may now be in dire financial straits and will not still be there. I do hope the guidance is safely amended or we may well face the decimation of the rich amateur musical scene in our country, which would be a tragedy. [Female; 25-34; Member; All; Qu 53]*  *Our choir has unfortunately had to stop and now are focussing on corporate gigs to make sure they can keep running as a business.[Female; 25-34; Female; Member;MT; Q53]*  *Our choir really misses not being able to sing together in our normal rehearsals. Frustration that we cannot raise money for charities from our concerts. [Female; 65-74; Facilitator; Live T; Qu53]*  *I cancelled my last live session right before lockdown as things were just getting more serious and I began to feel overwhelmed and decided I needed the space for my own mental health, to decide a plan of action.I was gutted and it sparked something in me - and I designed my first virtual choir project that same night, before serious lockdown was announced. I also ‘owed’ some sessions to members who had Pre paid for the term, and didn’t want to refund them so had to keep something going. Also. I was stubborn and didn’t want it to get the better of everything I had built up over the past 5 years! [Female; 35-44; facilitator; All; Qu53]* |
|  | Responsibility and loyalty | *I refused to let the singing die - It was important for some choir members to have that time of the week of meeting and singing together [Male; 35-44; Facilitator; MT and Live T; Qu 44]*  *Would like to stop participating in Zoom rehearsals but feel obliged to continue for loyalty's sake. Am really quite miserable about it.’ [Female; 65-74;Member; Live T; Qu 53]*  *I felt a sense if (sic) responsibility to each choir to continue to provide content and create social get togethers remotely’ [Female; 45-54; facilitator; Live T; Q 44]*  *I get more out of the SFTBrain singing group online. Enjoy seeing those needing this support.Sometimes don't feel like " bothering " to join the community choir zoom group - but usually do out of loyalty - I am a founder member and have been in that choir for more than 10 years. [Female; 65-74; member; Live T; Q53]* |
|  | Motivation and engagement | *There is a lack of motivation with the return to active singing seeming to be months away though we are all trying to learn three or four new songs whilst away [Male; 65-74; Member; None; Qu53]*  *Initially, the community was holding together but I fear with time, enthusiasm may fade. Concerts are being postponed but with no idea how long for it is hard to maintain motivation to learn the music. [Female; 45-54; Member; Live T; Qu 53]*  *My members have found online helpful and it has given them motivation and something to look forward to, I have found it hard and frustrating, but have continued to do it because it has helped so many ladies in this difficult time. They haven’t even wanted to stop for the summer! [Female; 55.64; Facilitator; Live T; Qu 53]*  *It is good to have our usual chat with other members - we break out into chat rooms by voice which reflects our normal pattern. However there are some members who have not engaged in the on line version of the choir at all which is concerning as they are mainly the older members of the choir. [Female; 55-64; Member; Live T; Qu53]*  *It has been hard keeping up the momentum, even though I introduced weekly online Zoom sessions, and a daily music quiz. I am convinced that a reasonable percentage of the membership will be reduced by the time we are allowed back indoors to rehearse. [Female; 55-64; facilitator; Live T; Qu53]* |
| **Wellbeing** | Wellbeing | *To support my choir members' mental and emotional health, keep them connected [Female; 55-64; facilitator; Mt and Live T; Q 44]*  *Music rehearsals are like a kind of meditation - they push all life’s daily stresses away and rejuvenate you. I hadn’t realised what a big part collective music played in maintaining my balance/wellbeing until it was removed. You simply cannot get the full experience by singing into your computer against a click track [Female; 35-44; Member; MT; Qu 53]*  *Face to face interaction is sometimes lost, connection issues WiFi ect, doesn’t give you same wellbeing experience as you would if you were singing together [Female; 35-44; Member; Live T; Q 24]*  *Its (sic) keeping the choir together and is good for my mental health. [Female; 55.64; Member; All; Q23]*  *Now its gone I'm realising just how much choir affected my mental and spiritual well-being, and how proud I was of what we produced. our Choir directors have been amazing at trying to keep us together but it's felt like quite a labour. [Female; 35-45; Member; MT and Live T; Qu 53]*  *I would rather have this opportunity than nothing. Singing is my therapy. It is something I could not imagine doing without [Female; 65-74; Member; MT and Live T; Qu23]*  *We managed to raise £5000 for a charity with our virtual choir performance so that was good. But basically my mental health has crashed and with no idea when we might be able to get together again I am losing hope. [Female; 45-54; Facilitator; All; Qu53]* |
|  | Sense of purpose | *Quire singing is an important part of my life; it helps me feel alive, so not being able to practise together properly is quite destructive of my overall sense of wellbeing*  *It is very important for people's mental health to keep connected. It is not much good being healthy and avoiding Covid-19 if you are suffering from severe depression because you have lost touch with people. The choir is an important lifeline in this. [Female; 65-74; Member;Mt and Live T; Qu 53]*  *Its (sic) keeping the choir together and is good for my mental health.*  *I suppose you can feel part of something bigger, which contributes to a sense of well-being [Female; 55-64; member; Mt and Live T; Qu23]*  *I didn't realise how important my choir was to my wellbeing and sense of identity. The choir is a community and support network, and it's very hard to be without it. [Female; 35-44; Member; All; Qu 53]* |
|  | Stress | *You feel isolated making the recordings which can be stressful for a chorus member who isn't used to and doesn't want to sing alone. [Female; 65-74; Member; MT and Live T; Qu 19]*  *Hard not to have another voice in your ear. No atmosphere can make it a little depressing [Female; 35-44; Member; All; Qu 22]*  *Without the virtual choir, I would have found it much harder. The singing helped keep me on an even keel, I felt more relaxed after each session. [Female; 55-64; MT and Live T; Qu53]*  *I sing in choirs for the sheer joy of making music with other people. The concept of doing this over zoom is horrific, unmusical, and just makes me sad for what I’m missing! I tried one online video recording and hated every second. It was frustrating, exhausting and just made me sad! [Female; 35-44; Member; None; Qu 26]*  *I really wanted to join in with online choirs but then the idea of singing alone to a screen really emphasised just how to much life had changed due to covid. So it made me sad rather than happy. [Female; 65-74; Member; None; Qu 26]*  *did not enjoy the experience, primarily because of the lack of instructions and my lack of knowledge. I found it stressful when usually I love singing. The final version of our first multi-track is not yet completed. [Female; 65-74; Member; Mt and Live T; Q19]* |
|  | Loneliness | *It's lonely and obviously difficult to have a realistic sense of the ensemble. [Male; 65-74; Member; MT; Qu 24]*  *Haven't been inspired by the music. Feels a bit lonely. [Female; 65-74; Member; None; Qu 26]*  *It can feel a bit lonely, especially when you say goodbye and click 'leave' And I'm singing alto on my own, which is weird (and freaks the cats out!) [Female; 55-64; Member; Live T; Qu 24]*  *can't hear other voices. Lonely/lack of social interaction. Feel my voice sounds awful [Female; 55-64; Member; MT; Qu 19]*  *The experience is still lonely and 50% of the choir did not want to or could not take part [Female; 55-64; facilitator; Mt and Live T; Qu 103]* |
| **Social Aspects** | Social contact | *only seeing others who may be behind you under normal practice conditions and more social interaction [Male; 65-74; facilitator; Live T; Qu 52]*  *We tried singing only once, and it was a disaster because of everyone's different bandwidths, so we now just use it as a social occasion. [Female; 55-64; facilitator; MT; Live T; Qu 39]*  *The main advantage is that it allows for a social occasion during these difficult times. The other advantage is that you are at home and don't need to travel, but it would not be my experience of choice. I would prefer to travel and sing together with the other choir members. [female; 65-74; member; Live T; Qu 52]*  *That the community and connectivity of a choir does not ENTIRELY rely on in-person presence. The uplifting and social aspects of a choir (which are by far as important as singing itself) can in part be replicated by online / virtual sessions, although they don't replace that completely and I firmly believe singing together is SAFE and should be brought back soon. [Female; 45-53; Member; Live T; Qu 47]* |
|  | Being part of something | *The social aspect is much less online. The feeling of being part of something bigger than myself. The blending of voices together - helped through the vocal challenges. [Male; 55-64; Member; Live T; Qu 24]*  *It’s lonely and disconnected without a sense of community and belonging [Female; 65-74; Member; MT and Live T; Qu 24]*  *There is every disadvantage. You can't hear voices on either side of you. You feel exposed. If you are uncertain of the music you are not helped by being in the virtual choir. You have no real contact with other choir members and, speaking for myself, the experience certainly did not make me feel part of a group. [Female; 75-84; Member MT; Qu 24]*  *It gives us a real sense of belonging and being part of something [Male; 75-84; Member; MT and Live T; Qu 53]*  *Not entirely fulfilling but enjoy hearing/seeing finished performance and makes you feel Part of the choir again [Female; 65-74; Member; MT; Qu19]*  *being part of something so big and involving people from different (sic) vounties [Female; 45-54; Member; MT; Qu 23]* |
|  | Community | *My choir is my business and income but also my family. I’ve worked for twelve years to build our community and they needed me to provide consistency and continuity which gave them a feeling of some stability during the worst of lockdown. I didn’t miss a single day. I went straight to it on 16th March when social distancing began [Female; 45-54; Facilitator; MT and Live T; Qu 44]*  *It’s lonely and disconnected without a sense of community and belonging [Female; 65-74; Member; All; Qu 24]*  *It is keeping us together as a choir and also gives us time to talk together which we do not normally do [Female; 65-74; facilitator; MT and Live T; Qu 39]*  *Again, I don't organise the choir, however the organising team were keen to take it online so that we still had a community that people could join in with if they wished to. A huge part of the choir is the social element and tht (sic) was still, sort of, able to continue [Female; 34-43; facilitator; MT and Live T; Qu 44]*  *Although recording your part alone at home feels odd at first, the more you participate in, the easier it becomes. Online social groups help keep give the lone singer a sense of community. However, the experience can never, ever replace singing live with others! As a stop gap it offers an opportunity to learn new works and keep one’s vocal chords exercised. [Female; 65-74; Member; Live T; Qu 19]*  *I tried out several virtual choirs at the beginning of lockdown. If I was feeling down and listless I found that singing in a virtual choir rehearsal lifted my spirits and energised me. The Self-Isolation Choir in particular makes me feel I belong to a community of like-minded singers. [Female; 65-74; Member; All; Qu 53]* |
|  | Physical contact | *It's good but could not replace choir in person. You don't get the physical interaction of being with everyone else and you can't of course hear what everyone else is singing at the time of recording. Watching and listening to the recording just lacks what a recorded live performance does. [Female; 55-64; Member; All; Qu 19]*  *I found it stressful to manage the backing track and recording.I miss the joining together, the fellowship, the corrections and interruptions of our choir director and the sociability. The immediate feedback of how we sound completely outstrips the receiving of the mixed result. [Female; 65-74; Member; MT; Qu 19]*  *I miss meeting in person so much. Online was ok for the first few weeks but then my interest in it waned. [Female; 55-64; Member; Live T; Qu53]* |
| **Musical Elements** | Musical growth | *Developing every week, working on new material, maintaining voice, keeping a structure in place and space to sing [Female; 35-44; Member; All; Qu 52]*  *I am more aware how bad I sound, overall we are not making the usual level of progress and we don't have the incentive of preparing for an actual performance [Female; 55-64; facilitator; MT and Live T; Qu 46]*  *The community choir I run is as much about friendship and caring so we have maintained a high level of contact with songs to karaoke along with, new material to try out (words and music), some inspiration and ideas for music to listen to from members [ Female; 65-74; facilitator; Qu 53]* |
|  | Teaching and Learning | *Also there is a sense of finding new ways of doing things. And therefore finding new pleasures and new rewards. And it gives so many educational opportunities. And a chance to explore the music [Female; 65-74; Member; MT and Live T; Qu23]*  *You get familiar with other software, you get to evaluate what you sound and look like singing - such as enunciating words clearly and visually expressing emotions - it definitely increases your confidence and makes you practice as your voice alone is being recorded. The downside is definitely hearing the other voices at rehearsals but this is compensated by hearing the finished audio visual result which is something you don't get to see or hear, only the audience get that. [Female; 65-74; Member; MT; Qu 53]*  *We've had a varied programme learning new music and techniques rather than rehearsing one or two pieces for a concert [ Female; 65-74; Member; Live T; Qu23]*  *I miss singing with others and the help that other choir members give when we are singing together. But it has been good to keep singing, learn new material and "see" friends on the screen. [Female; 55-64; Member; All; Qu 21]*  *no togetherness feel, no support from stronger singers, lack of learning opportunity or expanding repetoire (sic). [Female: 55-64; member; MT and Live T; Qu 24]* |
|  | Vocal skill | *It is very exacting- you have to face up to your imperfections and challenge your bad habits [Female; 55-64; Member; MT and Live T; Qu 23]*  *No need to travel, so takes less time out of the day. Becoming a better individual singer because I am singing individually and more aware if I sing the wrong note, run out of breath [Male; 45-54; Member; Live T; Qu 23]*  *For me I have developed voice craft techniques which have helped the quality of my voice [Female; 65-74 ; MT and live T; Qu 23]*  *I am making the best of things but would much rather be with my choir friends every week and performing at events, raising money and singing to an audience. This on-line choir life helps me to continue to sing regularly and improve my technique, thanks to You Tube sessions, recorded by our Director. Just keeping the faith until we’re able to sing together again. [Female; 65-74; Member; MT and Live T; Qu53]*  *Useful opportunity to reflect on style of singing and which direction to go in once singing eventually resumes - probably mid 2021 in reality. Time in between used to improve technique - primarily breathing and mouth / throat cavity shape*. [*45-54; unknown; member; MT; Qu 53]* |
|  | Musical skill | *Getting lost in another section’s part [Female; 65-74; Member; MT and Live T; Qu 25]*  *to learn your individual parts, when recording you hear your mistakes and wrong notes, so it is a good opportunity to work on improving intonation, notes etc [Female; 55-64; member; MT and Live T; Qu 23]*  *Developing members (sic) musical ability by targeted feedback is not possible if you can’t hear them. This is a big part of our normal live group rehearsal. [Female; 45-54; facilitator; MT and Live T; Qu 46]*  *I am more aware how bad I sound, overall we are not making the usual level of progress and we don't have the incentive of preparing for an actual performance[Female; 55-64; Member; Live T; Qu 24]* |
|  | Accuracy | *You don't always know if you are singing accurately. You can't always ask a question when you need to. Less social enjoyment. [Female; 25-34; Member; Live S; Qu 24]*  *It is strange sitting in your own room singing by yourself and clearly singing is such a collaborative thing in a choir.I can see it wouldn't be for everyone. Some people struggled with the idea of a virtual choir and the technology and just not singing with other people. Some people didn't like the fact they couldn't pitch their part well which is very valid and the reassurances you get with singing together in person. [Female; 55-64; Member; Live T; Qu 24]*  *You are singing to yourself the Musical director can't hear you so you don't get back any feedback wether (sic) you are singing correct or flat. [Male; 75-84; Member; MT and live T; Qu 21]*  *No feedback on your actual part and how you are singing and unable to hear how the whole choir sounds together [Male; 55-64; Member; Live T; Qu 24]* |
|  | Musical Confidence | *Singing alone to a backing track increase's (sic) one's self-confidence, and stops you relying on others to lead [Female; 35-44; MT and Live T; Qu 53]*  *You are forced to identify with your own tunefulness, and not rely on other singers to cover you when you go wrong [Female; 55-64; Member; MT and Live T; Qu 53]*  *Not meeting up with the choir and singing has greatly affected my mental health. It has shown me how much singing has helped me in the past. I need the choir around me in order to have the confidence to sing out. On my own I think I sound awful [Female;55- 64;Member; MT and Live T; Qu 53]*  *you can sometimes sing like no one can hear you and it is liberating [Female; 45-54; Member; All; Qu 23]*  *singing alone is not always something I have the confidence or ability to do [Female; 65-74; Member; None; Qu 53]*  *Lost the will to go back. No confidence in singing online with headphones. Online choirs expose my inabilities and the lower standard than I thought of the choirs I'm in [Male; 55-64; Facilitator; None; Qu 53]* |
| **Co-creation through Singing** | In the moment | *I didn't like the way my voice sounded on its own, but I sent in my recording anyway, putting faith in the choir leader to blend it all together nicely - and she did! [ Female; 34-44; Member; Qu19]*  *Live and co-present group singing, in four part harmony in our case, is not replicable online. I miss the sensation dreadfully [Non-binary; 45 - 54; Member; both; Qu 24]*  *When recording my part it felt less enjoyable and far more pressurised. Felt very exposed and was relieved when I saw/heard the final thing and all voices had been blended together [Female; 55-64; Member; All; Qu 19]*  *I really miss singing with others, how our voices blend in person, etc. But I have to say that the final products have all been really amazing! [ Female; 45 - 54; Member; Both, Qu 19]*  *It's nice to hear the blend and feels more rewarding and gratifying than the other choir experience of learning songs over zoom [Female; 35-44; Member, Both, Qu 19]* |
|  |  |  |
|  | Magic | *The experience lacks a certain magic and feedback that you can only gain from a liver (sic) performance [Female; 55-64; Member; Both; Qu 19]*  *It’s completely different, and doesn’t allow me to experience the joy of singing with other people. The technicalities block the magic for me [Female; 45-54; Member; both; Qu 21]*  *What's missing is the thrill of hearing my voice blend with others and being able to respond to the conductor and to the sound I hear. [Female; 55-64; Member; Both; Qu 19]*  *For me group singing is an immersive experience, being aware of and interacting with folk around, and involves picking up subtle cues by a musical sixth sense that is not present in the clinical, sterile world of virtual attempts to produce a pseudo-choral experience. [Female; 65-74; Member; none; Qu 26]*  *When we sing we are creating something even greater than the sum of its parts, something entirely ephemeral that we hone and improve until our breath becomes a knock on the door of the soul. I'm not always very good at talking to others but when we sing together we create bonds that are no less strong for their being almost subconscious. I know which of my fellow 2nd Altos I can rely on in different areas of music, I know which 1st Altos will duet properly if I stand next to them, I know which Basses I can rely on to keep me in pitch and which Tenors will project a subtle opening from the other side of the room so that I can come in on time. This is what we are cut off from, what we are losing. [Female; 25-34; Facilitators; None; Qu53]* |
|  | Musical Cohesion** | *At the start of lockdown, I participated in a couple of Zoom online choirs, one from the choir I am a singer in, and one other. I was shocked at how limited and dispiriting this felt, in contrast to being surrounded by 4 part harmony with friends…... Now, 17 weeks down the line, I'm able to sing, and listen to glorious music, but am still deeply, profoundly missing the glorious, all-embracing joy of singing with others. [Female 35-44; Member; Live; Qu 24]*  *As a facilitator, I have really missed 'real' faces and positive feedback. The technology has worked for the most part, but it is worrying and frustrating when it doesn't work properly. We all miss the powerful swell of harmony that is experienced in a room with a large group of singers. This cannot be achieved via a zoom choir and nothing can replace the benefits, sense if achievement and wonder of it.*  *Multi-Track feels a little more like a group of soloist mashed together, rather than a blend of choral voices [ Female; 35-44; Member; All, Qu 19]*  *It is hard being alone with my voice - I miss being able to hear (and blend with) my fellow singers [Male 55-64; Member; MT and Live, Qu 21]*  *At the start of lockdown, I participated in a couple of Zoom online choirs, one from the choir I am a singer in, and one other. I was shocked at how limited and dispiriting this felt, in contrast to being surrounded by 4 part harmony with friends…... Now, 17 weeks down the line, I'm able to sing, and listen to glorious music, but am still deeply, profoundly missing the glorious, all-embracing joy of singing with others. [Female 35-44; Member; Live, Qu 21]*  *Much of the choral experience is the blending and interaction of voices in real time [ Male; 65-74; Member; Live; Qu 21]*  *Not same sense of unity and nothing like the surround sound and blend of singing as a group [Female; 45-54; Member; Live; Qu 21]*  *Being part of a choir you listen to each other and blend voices which is not possible in the virtual world [Female; 55-64; Member; Live]*  *Choirs develop by the members singing together in a real acoustic and adjusting to what each singer hears. This can only be done in 'real time' and in person. By removing this person-to-person contact the essential nature of choir music making is changed, and usually degraded [Male; 75-84; facilitator; Live T; Qu 53]*  *It seems that there is commonality to the grief and loss that singers feel when they are unable to sing in choirs. It underlines the extent to which singing / harmonising with others have very positive benefits on the wellbeing [Female; 65-74; member; All; Qu 53]* |
|  | Physicality | *You don't get the physical interaction of being with everyone else and you can't of course hear what everyone else is singing at the time of recording [Female; 55-64; Member; Both; Qu 16]*  *you are not physically with people, so you are not interacting listening to other sops or altos to blend, helping each other [Female; 55-64; Member; Live T; Qu 21]*  *The thing that is missing is the physical sense of the group [Female 65-74; Member; Both; Qu 21]*  *I miss blending with people in real life and feeling that sense of connection [Non-binary; 35-44; Member; Both, Qu 21]*  *in a real choir you feel a physical and emotional connection with the interweaving and blending of part which is totally missing Qu24*  *There is something very special about singing in a physical group, than (sic) cannot be replicated via the internet… I struggle to quantify what this special thing is. As much as I enjoy being able to still see my choir friends and participate in rehearsals over the internet, there is definitely something missing which does have a detrimental effect.[Female; 25-34; Member; Both, Qu 24]*  *Singing physically with other people is very morale boosting and the social interaction is vital for my wellbeing.[Female; 75-84; Member; Live T; Qu 24]*  *It's not the same at all - the vibrations and the personal interactions are missing [Female; 65-74; Member; Live T; Qu 21]* |
|  |  |  |
|  | Emotional connection | *The social and emotional well-being of singing together in person, bouncing off the energy of one another, listening to each other and breathing are all lost when using Zoom or pre-recording. [Female; 25-34; Member; Both; QU 24]*  *I wasn’t confident I was singing well as I couldn’t blend my voice. I didn’t get the same buzz [female; 35-43; Member; MT; Qu 19]*  *It is not a comparable experience and much less able to improve your singing and feel the emotion and power if (sic) a collective voice. [Female; 45 -54; Member; Live; Qu 24]*  *it is not at all fulfilling, whereas singing together in a choir in reality gives a buzz, relaxes you, it is enjoyable.[Female; 65-74; Member; All;Qu 24]*  *No shared experience; can't hear the other parts; very boring when the conductor runs through every part's line, can't sing well seated; poor acoustic in my study; only hear myself; poor sound quality from my computer; if you use Zoom at work then it feels like being at work; in a real choir you feel a physical and emotional connection with the interweaving and blending of part which is totally missing [Female; 45-54; MT; Q59]* |
|  | Audience | *Whilst it is satisfying to record something at home and see it finally edited together with everyone else’s video nothing beats the physicality and sociability of singing, in time, together. [Female 45-54; Member; Both; Qu 21]*  *I really miss singing with others, how our voices blend in person, etc. But I have to say that the final products have all been really amazing! [ Female; 45 - 54; Member; Both, Qu 19]*  *difficult to perform for an audience and no real "buzz" from it [Female; 55-64; Member; Live; Qu 24]*  *No live performances, although we have had a session on FB Live as part of a virtual festival. MUCH LESS FUN! [Male 55-64; Member; Live T; Qu 24]*  *[lack of] Audience participation and reaction [Female; 35-44; Member; MT; Qu 19]* |
